# Gene discovery by genome-wide CDS re-prediction and microarray-based transcriptional analysis in phytopathogen *Xanthomonas campestris*

**DOI:** 10.1186/1471-2164-12-359

**Published:** 2011-07-12

**Authors:** Lian Zhou, Frank-Jörg Vorhölter, Yong-Qiang He, Bo-Le Jiang, Ji-Liang Tang, Yuquan Xu, Alfred Pühler, Ya-Wen He

**Affiliations:** 1 National Center for Molecular Characterization of GMOs and State Key Laboratory of Microbial Metabolism, School of Life Science and Biotechnology, Shanghai Jiao Tong University, Shanghai 200240, China; 2 Universität Bielefeld, CeBiTec, Universitätsstr.25, D-33615 Bielefeld, Germany; 3 State Key Laboratory for Conservation and Utilization of Subtropical Agro-bioresources, Nanning 530004, China

**Keywords:** *Xanthomonas campestris*, CDS re-prediction, microarray analysis, new CDS

## Abstract

**Background:**

One of the major tasks of the post-genomic era is "reading" genomic sequences in order to extract all the biological information contained in them. Although a wide variety of techniques is used to solve the gene finding problem and a number of prokaryotic gene-finding software are available, gene recognition in bacteria is far from being always straightforward.

**Results:**

This study reported a thorough search for new CDS in the two published Xcc genomes. In the first, putative CDSs encoded in the two genomes were re-predicted using three gene finders, resulting in the identification of 2850 putative new CDSs. In the second, similarity searching was conducted and 278 CDSs were found to have homologs in other bacterial species. In the third, oligonucleotide microarray and RT-PCR analysis identified 147 CDSs with detectable mRNA transcripts. Finally, in-frame deletion and subsequent phenotype analysis of confirmed that Xcc_CDS002 encoding a novel SIR2-like domain protein is involved in virulence and Xcc_CDS1553 encoding a ArsR family transcription factor is involved in arsenate resistance.

**Conclusions:**

Despite sophisticated approaches available for genome annotation, many cellular transcripts have remained unidentified so far in *Xcc *genomes. Through a combined strategy involving bioinformatic, postgenomic and genetic approaches, a reliable list of 306 new CDSs was identified and a more thorough understanding of some cellular processes was gained.

## Background

Over the past two decades, we have witnessed the publication of more than 1,000 complete microbial genome sequences (http://www.ncbi.nlm.nih.gov/genomes/). The trend towards genome sequencing is expected to continue or even accelerate in the near future. The wealth of sequence information has greatly enhanced our understanding of bacterial physiology and biological processes underlying the very organization of life. One of the major tasks of the post-genomic era is "reading" genomic sequences in order to extract all the biological information contained in them. An essential step in this quest is the identification of protein-coding genes, with subsequent functional annotation of the corresponding gene products [1]. A number of gene-finding methods have been developed to address this problem from different points of view. Generally, these gene-finding methods are divided into two broad categories [2]. "Extrinsic" methods take into account information derived from similarity search procedures [3]. "Intrinsic" methods, which deal with DNA sequence only, use statistic or pattern recognition algorithms to find genes in DNA through detection of specific motifs or global statistical patterns. For example, GeneMark employs a hidden Markov model (HMM) to find genes [4-6] while GLIMMER employs an interpolated Markov model [7-9]. Although a wide variety of techniques is used to solve the gene finding problem and a number of prokaryotic gene-finding software are available, gene recognition in bacteria is far from being always straightforward and there are still a lot of wrong or inaccurately annotated genes and missing genes in the published genomes [1,10-14]. A major reason for this situation may be that genes can be tightly packed in prokaryotes, resulting in frequent overlap. Thus, detection of translation initiation sites and/or selection of the correct coding regions remain difficult [1]. In addition, it is now well known that all microbial genomes contain an abundance of short genes [11,15]. For statistical reasons, the longer the sequences, the easier it is to detect the codon bias. The short length of these genes probably affects both pillars of CDS prediction, namely intrinsic and extrinsic approaches [11,16].

The *Xanthomonas *genus is one of the most ubiquitous groups of plant-associated bacterial pathogens. Members of this genus have been shown to infect at least 124 monocotyledonous and 268 dicotyledonous plant species [17]. *Xanthomonas campestris *pv. *campestris *(Pammel) Dowson (*Xcc *hereafter) is the causal agent of black rot of crucifers, which is possibly the most important disease of crucifers worldwide [18]. So far, genomes of the three *Xcc *strains ATCC 33913, 8004, and B100 have been sequenced [14,19,20]. The genome of *Xcc *strain ATCC33913 comprises a circular chromosome of 5,076,187 bp encoding a total of 4181 predicted CDSs [19]. The genome of *Xcc *strain 8004 resides on a single circular chromosome of 5,148,708 bp, which encodes 4273 predicted CDSs [20]. Although the majority of the genes encoded by the two genomes were identical, a total of 108 and 62 CDSs unique to *Xcc *8004 and *Xcc *ATCC33913 were respectively identified [20]. In particular, analysis of the genome of *Xcc *strain 8004 identified a total of 87 CDSs that have homologs in *Xcc *ATCC33913, but were not annotated by da Silva et al. [19]. Similarly, annotation of the recent sequenced genome of *Xcc *B100 identified more than 200 additional CDSs that were not annotated in the other two *Xcc *strains [14]. Although these newly identified CDSs need to be further verified, the findings suggest that there is still room for improvement in the state of gene identification of *Xcc *genomes.

In this study, putative protein coding sequences in the two genomes of the *Xcc *strains 8004 and ATCC33913 were re-predicted using the latest version of three gene-prediction programs. A total of additional 2850 putative new CDSs were identified. Based on the results of similarity searching, transcriptional pattern analysis and functional analysis, a reliable list of 306 new CDSs was obtained from this data set. The function of two newly identified genes was further confirmed by gene deletion and subsequent phenotype analysis.

## Results

### CDS re-prediction and identification of putative new CDSs

**Figure 1:**
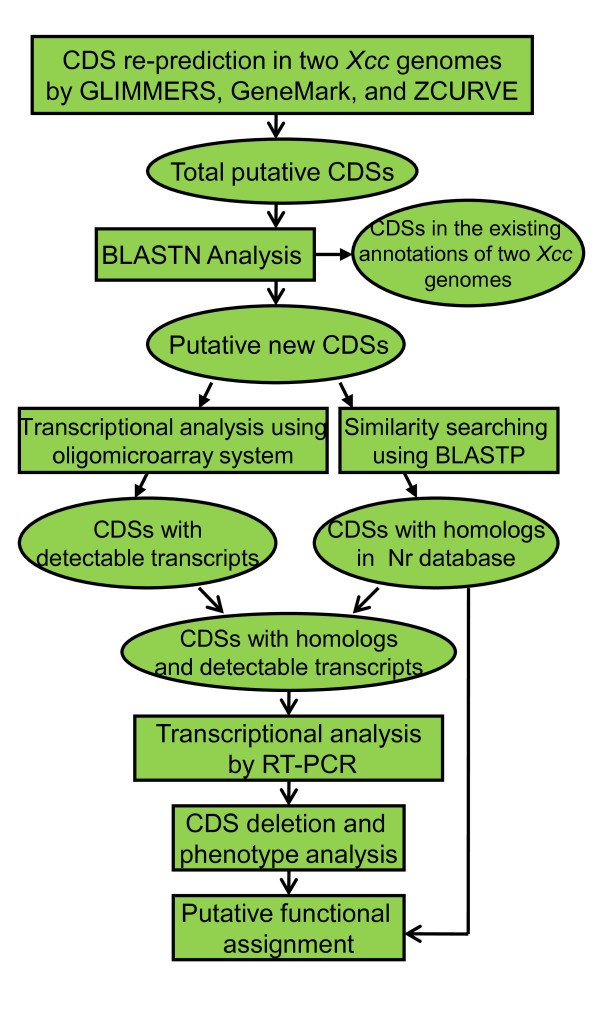
**Overall strategy for the identification of new CDS in *Xcc***.

**Figure 2:**
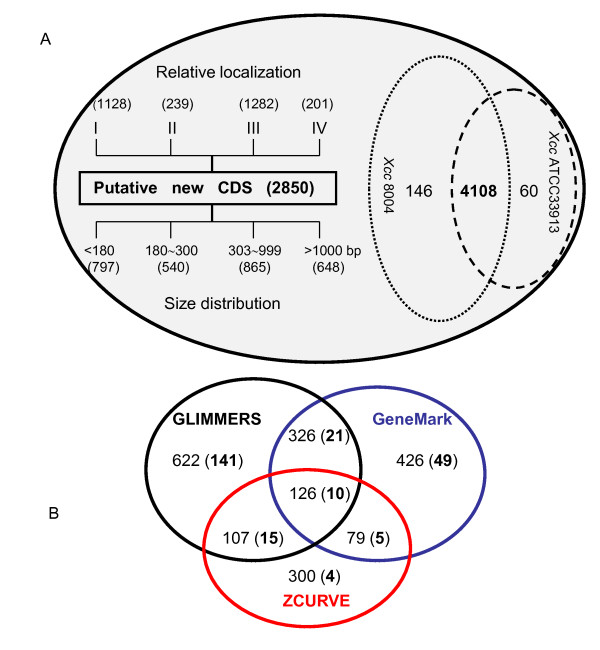
**Total CDSs identified in the genomes of *Xcc *strains 8004 and ATCC33913**. (A) The location of all putative new CDSs relative to existing neighbor CDSs on the chromosome of *Xcc *strain 8004 has been classified into four groups, indicated by I, II, III, and IV respectively on the left. Group I indicates intergenic regions on the coding strand; II indicates intragenic regions on the coding strand, CDSs are partially or fully overlapped with annotated CDSs, but they are in different reading frames; III indicates intergenic regions on the complementary strand; and IV indicates intragenic regions on the complementary strand, CDSs are partially or fully overlapped with annotated CDSs, but they are in different reading frames. Below the relative localization, the length distribution of the putative new CDSs is given in base pairs (BP). Numbers inside the brackets indicate the number of CDSs. (B) A VENN diagram showing the overlapping of the CDSs predicted by GLIMMER, GeneMark, ZCURVE, respectively. Numbers inside the brackets indicate the number of the CDSs that have been confirmed by extrinsic evidence and/or transcriptional analysis.

In this study, by using a combined strategy (Figure 1) that the three well-established gene finders GLIMMER (http://cbcb.umd.edu/software/glimmer) [8], GeneMark [21], and ZCURVE [22] were respectively applied to predict putative protein coding sequences (CDSs) within the two genomes of *Xcc *strains 8004 and ATCC33913 [19,20], a total of 7164 CDSs were identified after further sequence analaysis (Figure 2). Among them, 4,314 CDSs, including 146 *Xcc *strain 8004-specific CDSs, 60 ATCC33913-specific CDSs and 4108 shared CDSs between the two genomes, have been previously annotated (Figure 2A). The remaining 2850 predicted CDSs have not been identified in the published two genomes and were defined as putative new CDSs (Figure 2A), including 1181 CDSs by GLIMMER, 957 CDSs by GeneMark, and 612 CDSs by ZCURVE (Figure 2B). Intriguingly, there were only 126 overlapping CDSs predicted by all the three gene finders (Figure 2B). The size of these putative CDSs ranged from 90 to 4545 bps, and most of them (2202 of 2850 CDSs) were less than 1 kb long (Figure 2A). In particular, 797 CDSs were less than 180 bp in length. BLASTN analysis revealed that 2410 of the 2850 putative new CDSs were located at intergenic regions of both strands (Figure 2A, indicated by "I" and "III") in the chromosome of *Xcc *strain 8004 (Figure 2A). The remaining 440 CDSs were partially or fully overlapped with the annotated genes, but within different reading frames (Figure 2A, indicated by "II" and "IV"). All of 648 putative new CDSs >1000 bp in length were either antisense or overlapping to the annotated genes in two *Xcc *genomes (Additional file 1).

### Validation of new CDS by extrinsic evidence

The set of 2850 putative new CDSs was probably contaminated by pseudogene fragments and false-prediction artifacts because all the 3 gene finders are entirely based on intrinsic evidence. To find true CDS, the next strategy used in this study was to get support by extrinsic evidence. All the putative new CDSs were blasted for similar entries within the NCBI non-redundent database by means of BLASTP. Based on the three criteria described in Materials and Methods, a total of 220 putative new CDSs were found to be significantly similar to other protein sequences in the database (Additional file 1).

More recently, the genome sequence of *Xcc *strain B100 has been published and the genome contained 496 additional CDSs [14]. About half of the these CDSs that were identified by the combined use of the gene finders GISMO [23] and REGANOR [24] were also present in the genomes of *Xcc *strains 8004 and ATCC33913, but have not been annotated [14]. Comparing the 2850 putative new CDSs identified in this study with the 496 additional CDSs in *Xcc *strain B100, we found an overlapping 72 CDSs (Additional file 1). Among them, 14 CDSs had more than one homologs in non-redundant database and have been included in the 220 putative new CDSs identified by similarity searching; the remaining 58 CDSs had no homologs in non-redundant database except in *Xcc *strain B100 and were also regarded as new CDSs in this study (Additional file 1). Taken together, a total of 278 CDSs were screened out of 2850 putative new CDSs by extrinsic evidence.

**Figure 3:**
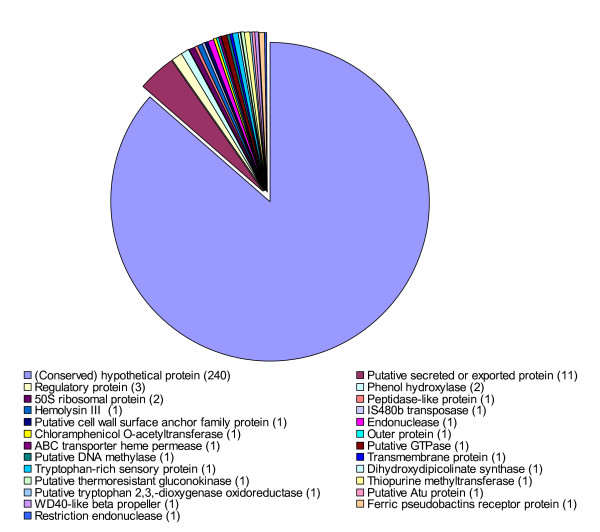
**Functional classification of the new CDSs based on similarity searching**.

The majority of these CDSs (240 of 278) encodes conserved hypothetical proteins or hypothetical proteins (Figure 3). Eleven CDSs (*Xcc*_CDS105, *Xcc*_CDS107, *Xcc*_CDS411, *Xcc*_CDS1381, *Xcc*_CDS1831, *Xcc*_CDS2249, *Xcc*_CDS2324, *Xcc*_CDS2391, *Xcc*_CDS2668, *Xcc*_CDS2723, *Xcc*_CDS2777) encode putative secreted or exported proteins and three CDSs encode regulatory protein or transcription factors (Figure 3). Xcc_CDS002 encodes a Sir2-like transcriptional silencer protein; Xcc_CDS1553 encodes an ArsR family transcriptional regulator; Xcc_CDS1633 bears similarity to the Homeodomain of POU domain proteins or HTH_XRE domain proteins (Additional file 1). Two CDSs (*Xcc*_CDS2171 and *Xcc*_CDS2691) encode putative phenol hydroxylases and another 2 CDSs (*Xcc*_CDS2201 and *Xcc*_CDS2211) encode putative 50S ribosomal proteins. The remaining 20 CDSs respectively encode peptidase-like protein (*Xcc*_CDS073), hemolysin III (*Xcc*_CDS095), IS480b transposase (*Xcc*_CDS177), putative cell wall surface anchor family protein (*Xcc*_CDS346), endonuclease (*Xcc*_CDS528), chloramphenicol O-acetyltransferase (*Xcc*_CDS639), outer protein D (*Xcc*_CDS900), ABC transporter heme permease (*Xcc*_CDS1309), putative GTPase (*Xcc*_CDS1342), putative DNA methylase (*Xcc*_CDS1416), transmembrane protein (*Xcc*_CDS1446), putative tryptophan-rich sensory protein (*Xcc*_CDS1617), dihydroxydipicolinate synthase (*Xcc*_CDS1689), thermoresistant gluconokinase (*Xcc*_CDS1836), thiopurine methyltransferase (*Xcc*_CDS1899), putative tryptophan 2,3-dioxygenase oxidoreductase (*Xcc*_CDS2015), putative Atu protein (*Xcc*_CDS2546), WD40-like beta propeller (*Xcc*_CDS2674), ferric pseudobactins receptor protein (*Xcc*_CDS2714), and restriction endonuclease (*Xcc*_CDS2849) (Figure 3; Additional file 1).

### Transcription analysis for new CDS

**Figure 4:**
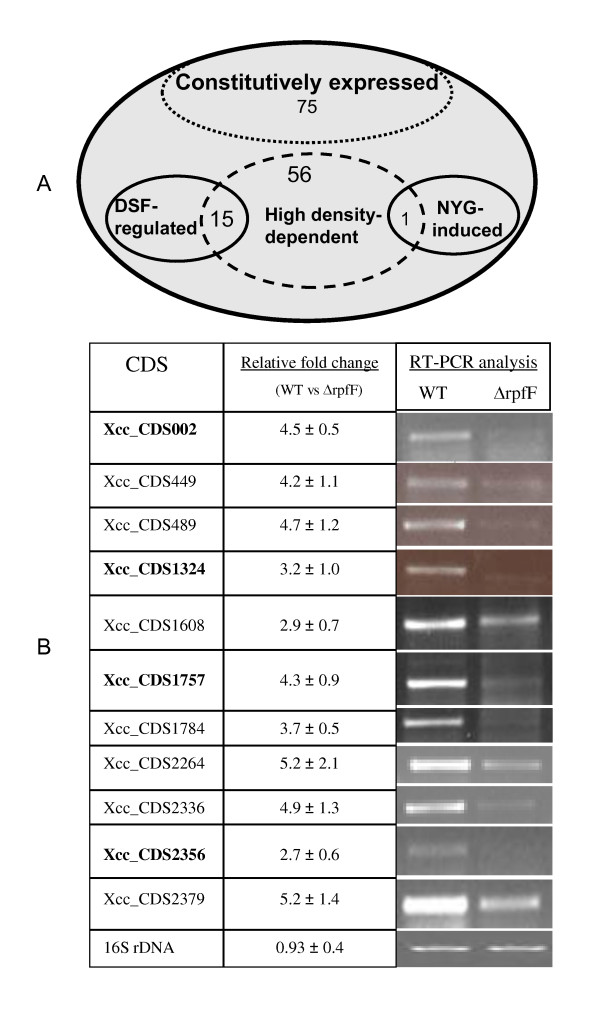
**Overview on new CDS identified via microarray and RT-PCR analysis**. (A) Transcriptional patterns of the new CDS based on microarry analysis. Constitutive expression indicates that these CDS are constitutively expressed at OD_600 _= 1.0, 1.6 and 2.0 during the growth. DSF-regulated expression indicates that the expression levels of these CDS at OD_600 _= 2.0 are significantly lower in ΔrpfF, ΔrpfC, or Δclp strains than those in wild type. NYG-induced expression indicates that this CDS is only transcribed at OD_600 _= 2.0 when grown in NYG medium. (B) RT-PCR analysis to verify the expression difference between the wild type and the *rpfF *deletion mutant ΔrpfF. The 4 new CDS supported by extrinsic evidence were indicated by bold font.

An alternative approach to validate a CDS is to detect the transcribed mRNA. An oligonucleotide microarray chip, which contains 50-mer oligos specific for 4080 annotated CDSs and 8 negative controls, has been successfully used to analyze the DSF regulon, Clp regulon and RavR regulon in *Xcc *[25-27]. In this study, a new microarray chip with the above-mentioned oligos and additional oligos specific for the 1724 putative new CDSs was constructed. This microarray chip was used to detect transcripts of the putative new CDSs. To detect transcripts under different conditions, total RNA was extracted from cell culture grown under the following conditions: (i) different cell density: OD_600 _= 1.0, 1.6 and 2.0; (ii) different genetic backgrounds: ΔrpfF strain, ΔrpfC, Δclp and ΔravR [25-27]; (iii) different media: rich YEB medium and poor NYG medium. By using the screening procedures described in Materials and Methods, 147 putative new CDSs were found with detectable transcripts (Figure 4A; Additional file 1). Further analysis revealed that 75 CDSs were constitutively expressed during the growth and the remaining 72 CDSs were only expressed at high cell density (OD_600 _= 2.0) (Figure 4A). Comparing the global gene expression profiles of *Xcc *wild type, *rpfF*, *rpfC*, and *clp *deletion mutants, we found that the transcription of 15 high cell density-dependent CDSs was also positively regulated by the quorum sensing signal DSF [25]. The expression levels of these CDSs in an *rpfF *deletion mutant were respectively 2.7 to 5.2 times lower than those in the wild type XC1 strain (Figure 4B). The transcription of *Xcc*_CDS2497 was only induced in poor NYG medium at higher density (OD_600 _= 2.0) (Figure 4A).

In order to go further in the validation of our microarray-based method for selecting true CDS, and as we are more interested in DSF signal-regulated CDSs, we chose the 15 DSF signal-regulated CDSs for further transcriptional analysis by reverse transcription PCR. The products of 11 CDSs could be amplified by using total RNAs extracted from cell culture at OD_600 _= 2.0 (Figure 4B). The resultant RT-PCR products were further verified by sequencing analysis (data not shown). RT-PCR analysis also verified the transcriptional difference of the 11 new CDSs between wild type and *rpfF *deletion mutant (Figure 4B).

### Total new CDSs identified by similarity searching and transcriptional analysis

**Figure 5:**
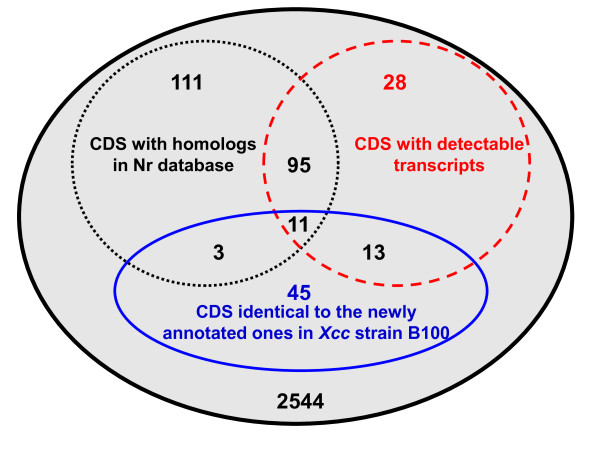
**Total new CDS identified by similarity searching and transcriptional analysis**.

While extrinsic evidence supported the presence of 278 new CDSs, and while transcriptional analysis indicated 147 new CDSs with detectable transcripts, a comparison of the two sets of new CDSs revealed a total of 119 overlapping CDSs that were identified by both approaches (Figure 5). Thus, a total of 306 (278+147-119) CDSs got support by extrinsic evidence or/and experimentally transcriptional analysis, suggesting that they are probably true CDSs. The remaining 2544 putative new CDSs failed to get support by extrinsic evidence or transcriptional analysis (Figure 5). Two of the overlapping 119 CDSs, Xcc_CDS002 and Xcc_CDS1553, which both encoded putative transcription factors, were chosen for further experimental characterization. The results are presented in the following sections.

### *Xcc*_CDS002 encodes a SIR2-like domain protein and is associated with virulence on Chinese cabbage

**Figure 6:**
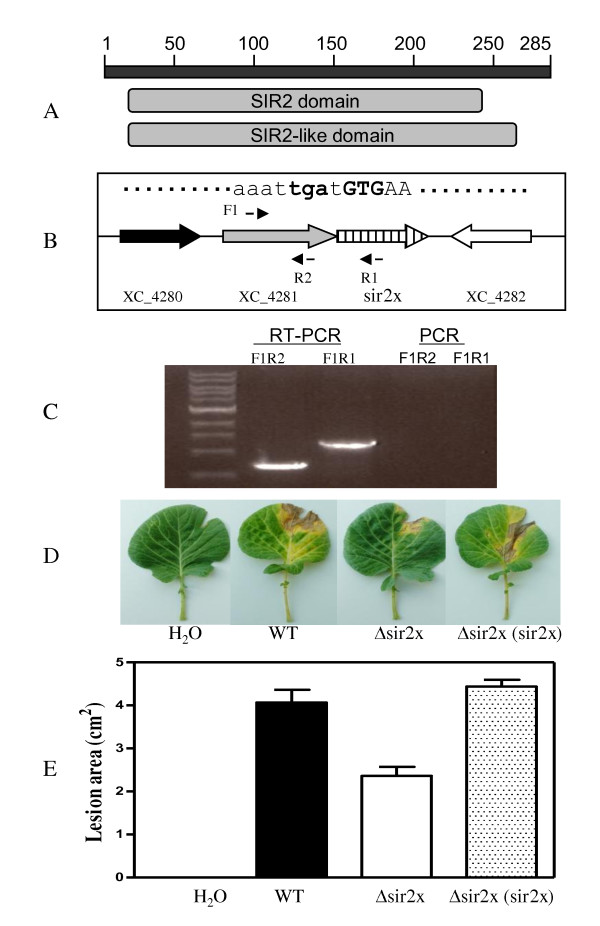
**The new CDS *sir2x *is involved in virulence in *Xcc***. (A) Domain organization of Sir2x as predicted by SMART (http://smart.embl-heidelberg.de/). (B) Genomic localization of *sir2x *and its flanking genes in the chromosome of *Xcc *strain 8004. (C) RT-PCR analysis of the XC_4281-*sir2x *operon. No genomic DNA contamination was indicated by normal PCR amplification using total RNAs as template. (D) *In vitro *virulence assay on Chinese cabbage. Δsir2x (*sir2x*) indicates the complemented deletion mutant defective in *sir2x*.

*Xcc*_CDS002 is a new CDS of 855 bps in length. It encodes a protein with a conserved silent information regulator 2 (SIR-2) or SIR2-like domain (Figure 6A), which has been found to confer NAD-dependent protein deacetylase activity in eukaryotes [28,29]. For the convenience of discussion, *Xcc*_CDS002 was renamed as *sir2x *for SIR2-like protein gene in *Xanthomonas campestris *in this study. The DNA sequence of *sir2x *was found in all the 3 published *Xcc *genomes and in *Xcc *strain XC1 (Figure 6C). In the genome of *Xcc *strain 8004, *sir2x *is flanked by XC_4281 and XC_4282 (Figure 6A), which respectively encode a phage-related regulatory protein cII and a hypothetical protein. *Sir2x *and XC_4281 share the same transcriptional orientation and are separated by only one base pair (Figure 6B). Further RT-PCR analysis confirmed that *sir2x *and XC_4281 are transcribed as an operon (Figure 6C). To further study its role in *Xcc*, the coding region (33 to 280 aa) of *sir2x *was in frame deleted in the chromosome of *Xcc *strain XC1 and the resultant mutant was named as Δsir2x. Deletion of *sir2x *did not affect the production of virulence factors, including extracellular protease, extracellular cellulase, and EPS (data not shown), but significantly reduced virulence of *Xcc *strain XC1 on Chinese cabbage (Figure 6D). Complementation of the mutant with the *sir2x *coding region resulted in the complete recovery of virulence to wild-type level (Figure 6E).

### *Xcc*_CDS1553 is associated with arsenate resistance in *Xcc *strain 8004

**Figure 7:**
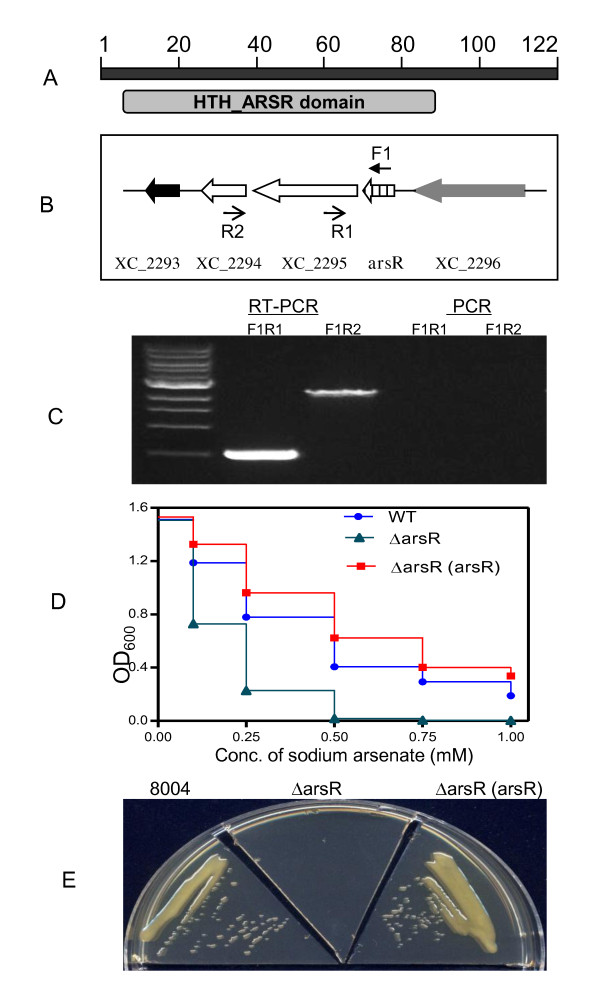
**The new CDS *arsR *is involved in arsenate resistance in *Xcc *strain 8004**. (A) Domain organization of *arsR *predicted by SMART (http://smart.embl-heidelberg.de/). (B) Genomic localization of ArsR and its flanking genes in the chromosome of *Xcc *strain 8004. (C) RT-PCR analysis of the *arsR*-XC_2295-XC_2294 operon. (D) Survival of *Xcc *strain 8004 and its derivatives in liquid NYG media with different concentrations of sodium arsenate. (E) Growth of *Xcc *strain 8004 and its derivatives on an NYG plate with 0.5 mM sodium arsenate. ΔarsR (*arsR*) indicates the complemented deletion mutant defective in *arsR*.

*Xcc*_CDS1553 encodes a 122-aa protein with a conserved HTH_ARSR domain (Figure 7A), which occurs in arsenical resistance operon repressors and similar prokaryotic, metal-regulated homodimeric repressors that belong to the ArsR superfamily of bacterial transcription-regulatory proteins [30,31]. For the convenience of discussion, this CDS was renamed as *arsR*. Interestingly, *arsR *was only found in the genome of *Xcc *strain 8004, not in *Xcc *strains ATCC33913 and B100. In the *Xcc *8004 genome, *arsR *is located upstream of XC_2295 and XC_2294, which respectively encode a putative high-affinity Fe^2+^/Pb^2+ ^permease and an arsenite efflux pump AcR3 (Figure 7B). *arsR *and XC_2295 were separated by 64 bps and the gap between XC_2294 and XC_2295 was 83 bps (Figure 7B; 20). Further RT-PCR analysis showed that *arsR*, XC_2295, and XC_2294 belong to the same operon (Figure 7C), suggesting that ArsR, XC_2294 and XC_2295 might be functionally related. To further confirm this hypothesis, an *arsR *in frame deletion mutant termed ΔarsR was generated in *Xcc *strain 8004. The results showed that the ΔarsR strain was much more sensitive to arsenate than the wild type strain (Figure 7D). On LB plates with 0.5 mM arsenate, the wild type strain *Xcc *8004 grew well, while in contrast, the deletion mutant did not grow at all on this medium (Figure 7E). The mutant phenotype could be reverted by complementation with a plasmid carrying the coding region of *arsR*, demonstrating that the observed phenotype was due to *arsR*.

## Discussion

In this study, we used a combined strategy for CDS prediction. GLIMMER is a computational gene-finding system and the technical underpinning of the system is an interpolated Markov model (IMM), a generalization of Markov chain methods [8]. The GeneMark program is an *ab initio *gene finder, which employs inhomogeneous (three-periodic) Markov chain models describing protein-coding DNA and homogeneous Markov chain models describing non-coding DNA [6]. ZCURVE is a system for recognizing protein-coding genes in bacterial genome, which uses the "Z-transformation" of DNA as information source for classification [22]. The results showed that 99.7% of the CDSs (4168 of 4181) in the existing annotations of strain ATCC33913 and 99.5% of the CDSs (4254 of 4273) of strain 8004 could be predicted by the combined strategy (Figure 2A), suggesting that the combined gene finding strategy works well for finding currently annotated genes in *Xcc *genomes. In addition to the CDSs in the existing annotations of *Xcc *genomes, a total of 2850 putative new CDSs were identified in the two *Xcc *genomes by the combined gene prediction strategy. Among them, 306 reliable new CDSs were further confirmed by subsequent analysis based on extrinsic similarity or/and transcript detection, suggesting that the combined gene finding strategy could be used for finding new CDS in bacterial genomes. Considering the number of putative CDSs predicted and those having been confirmed by extrinsic evidence and/or microarray analysis (Figure 2B), GLIMMER seems more powerful than GeneMark and ZCURVE in new CDS prediction.

Microarrays traditionally have been used to analyze the expression behavior of large numbers of annotated genes in bacteria. In this study, microarray analysis, applied together with CDS prediction, was used to find new genes, which was further validated by RT-PCR analysis. Compared to other transcript detection methods, microarray analysis is more sensitive and suitable for highthroughput analysis. So far, a similar strategy has only been reported for *Escherichia coli*. Selinger et al. [32] introduced a high-density oligonucleotide probe array for *E. coli *that not only carries strand-specific probes for all mRNA, tRNA, and rRNA regions, but also covers intergenic regions of >40 bp. Using *E. coli *RNA from cells grown on different media, over 1100 transcripts corresponding to intergenic regions were identified. Further classification revealed 317 novel transcripts with unknown function [33].

SIR2 proteins are found in organisms ranging from bacteria to humans [28]. In eukaryotes, SIR2 proteins regulate transcriptional repression, recombination, the cell division cycle, microtubule organization, cellular responses to DNA-damaging agents and aging [28,29]. A phylogenetically conserved NAD^+^-dependent protein deacetylase activity has been demonstrated in Sir2 family proteins in eukaryotes [34-36]. So far very limited evidence is available regarding the function of SIR-2 proteins in bacteria. The only reported case was from *Salmonella typhimurium*, where the gene cobB is involved in the biosynthesis of cobalamin and the catabolism of propionate [37]. Further analysis revealed that the recombinant SIR2 protein CobB had NAD-dependent ADP-ribosyltransferase activity *in vitro *[38]. The demonstration that the ribosyltransferase and NAD^+^-dependent protein deacetylase activities are both dependent on an acetylated substrate confirms the fundamental link between the two activities [29]. The true enzymatic activity of Sir2x and how Sir2x is involved in the regulation of virulence in Chinese cabbage remains to be dissolved. The involvement of *sir2x *in virulence of *Xcc *strain XC1 is in good agreement with previous findings that transposon insertion in the promoter region of XC4281 encoding a phage-related regulatory protein cII led to a complete loss of virulence of *Xcc *strain 8004 on radish [20]. As shown in Figure 6, XC4281 and the newly identified *sir2x *are within the same operon and they share a common promoter. Transposon insertion in the promoter region probably disrupts not only the expression of XC4281, but also the expression of *sir2x*. The roles of Sir2x in *Xcc *virulence remains to be dissolved.

Arsenic, a toxic metalloid, is currently and has always been ranked first on the Superfund List of Hazardous Substances (available on the World Wide Web), in part because of its environmental ubiquity. As a consequence, many bacterial species have genes that confer resistance to arsenic. Environmental arsenic is sensed by members of the ArsR/SmtB family of metalloregulatory transcriptional repressors [30,39], which represses the expression of operons involved in the uptake, efflux, sequestration, or detoxification of metal ions [40]. This study identified an ArsR family repressor and found that the XC2294-XC2295-*arsR *operon is involved in arsenate resistance in *Xcc *strain 8004. Since no ArsR homologs were found in *Xcc *strains ATCC33913, B100 and XC1, we propose that the *arsR *may have been acquired by *Xcc *strain 8004 in a lateral gene transfer event.

## Conclusions

This study reported a thorough search for new CDS in the two published *Xcc *genomes. In the first, putative CDSs encoded in the two genomes were re-predicted using three gene finders, resulting in the identification of 2850 putative new CDSs. In the second, similarity searching was conducted and 278 CDSs were found to have homologs in other bacterial species. In the third, oligonucleotide microarray and RT-PCR analysis identified 147 CDSs with detectable mRNA transcripts. Finally, in-frame deletion and subsequent phenotype analysis of the two newly identified CDSs confirmed their functionality. Our results showed that, despite sophisticated approaches available for genome annotation, many cellular transcripts have remained unidentified so far in *Xcc *genomes. Through a combined strategy involving bioinformatic, postgenomic and genetic approaches as demonstrated in this study, a reliable list of 306 new CDSs was identified and a more thorough understanding of some cellular processes was gained.

## Methods

### Bacterial strains and growth conditions

*Xcc *strains XC1 and 8004 were grown at 30°C with shaking (250 rpm/min) in YEB, LB or NYG medium as described by He et al. [25]. *E. coli *strains were grown at 37°C in LB medium. Antibiotics were added at the following concentrations when required: kanamycin, 100 μg/ml, rifampicin, 25 μg/ml, and tetracycline, 10 μg/ml.

### Nucleotide sequence source, gene prediction and domain analysis

Complete genome records of the *Xcc *strains ATCC33913 and 8004 [19,20] were downloaded from the NCBI Microbial genome database (http://www.ncbi.nlm.nih.gov/genomes/lproks.cgi?view=1). Gene prediction was conducted by the gene finders GLIMMER 2.03 [8], GeneMark [21] and ZCURVE [22]. For the prediction, the minimum length of CDS was set as 90 bp. BLASTN (http://blast.ncbi.nlm.nih.gov/Blast.cgi) was used to find the locations of all the putative new CDSs in the genomes of *Xcc *strain 8004 and ATCC33913. Multiple sequence alignment analysis was performed using CLUSTAL W (1.83) (http://sbcr.bii.a-star.edu.sg/clustalw/). Domain architecture analysis was performed using the SMART database application (http://smart.embl-heidelberg.de/). The nucleic acid sequences of two well-studied regulator *sir2x *and *arsR *have been deposited in the NCBI GeneBank database and the accession numbers are JF966390 and JF966391.

### Screening new CDS by extrinsic evidence

The amino acid sequences of all 2850 putative new CDSs were submitted for BLASTP analysis. Homologs in the nr database were selected on the basis of the following three criteria. Firstly, only the subjects with E-values lower than 10^-4 ^were considered hits. Secondly, the subjects should have similar sizes as the queries. Thirdly, for each query there should be more than one matched subject unless the E-value is very low (less than 10^-30^).

### Design and synthesis of CDS-specific oligonucleotides, and preparation of *Xcc *oligo microarray chip

Based on the annotated genome sequences of the *Xcc *strains ATCC33913 and 8004 [19,20], we used a CDS-specific oligonucleotide selection algorithm [41] to successfully design unique 50-mer oligonucleotides for 1724 putative new CDSs. The majority of these CDSs were more than 300 bps in length. As specificity controls, 50-mer oligonucleotides were also designed based on the sinat5 (NCBI No.: AF480944) and nac1 (NCBI No.: AF198054) genes of *Arabidopsis thaliana*, and the genes *rag1 *(NCBI No.: NM_131389) of zebrafish and the *olf1 *(NCBI No.:U56420) of *Homo sapiens *[25]. Thus, a total of 5770 CDS-specific oligonucleotides representing 4042 annotated CDS [25], plus 1724 putative new CDSs, and 4 specificity controls were used for the oligonucleotide microarray chip preparation. Oligonucleotides were synthesized at a 50 nmol scale by Operon Technologies (Alameda, CA, USA). The protocol employed for constructing the oligo-chip has been previously described [25]. Briefly, all oligos were dissolved in saline sodium citrate buffer (3 × SSC) to a final concentration of 40 μM. Oligo samples were arrayed with Pixsys 5500XL Arrayer (Cartesian) to poly-L-Lysine-coated microscope slides. DNA samples were fixed by rehydration, snap-drying and UV cross-linking. The remaining poly-L-Lysine on the slides was rendered non-reactive by treatment with blocking solution (150 mM succinic anhydride in 1-methy-2-pyrrolidinone, buffered with 85 mM sodium borate, pH 8.0) for 30 min. After washing with water, the array plates were rinsed with 95% ethanol and dried.

### Isolation of total RNA and microarray analysis

Bacterial cells were collected by centrifugation at 4°C for 5 min at 10,000 rpm. Total RNA samples were prepared by using RNeasy midi columns following the manufacturer's instructions (Qiagen). RNA integrity was confirmed by electrophoresis using a 1.3% formaldehyde agrose gel. The quality of DNA-free RNA was monitored by PCR and RT-PCR analysis of at least two known genes. Cy3- or Cy5-labeled cDNA was generated by using random hexamers as primers for reverse transcription (Invitrogen). cDNA labeling, purification and hybridization against the microarray were conducted as previously described [25]. Slides were scanned for the fluorescent intensity using a ScanArray 5000 laser scanner. The signal intensities were quantified by using the software ImaGene 5 (BioDiscovery). Hybridization signals were normalized using the scale normalization procedure previously described [25]. Each treatment was repeated three times and the data presented were the means of two representative replicates. The fold changes were then calculated from the normalized log ratios.

### Screening new CDS by statistical analysis of microarray hybridization signal intensity

In this study, oligonucleotide microarray analysis was used to detect transcription, so as to confirm the functionality of the putative new CDSs. The putative CDSs with detectable transcript was identified using the normalized signal median of the corresponding probe. To calculate the normalized signal median, firstly the average signal median S_0 _of 8 negative control probes representing 4 *Arabidopsis *and zebrafish genes [25] was determined by using the following formula: S_0 _= ∑(S_AZ_-B_AZ_)/8, where S_AZ _indicates the signal median of the negative control probe and B_AZ _indicates the corresponding background signal median. Secondly, the normalized signal median (S) of the putative new CDSs was calculated following the formula: S = S_CDS _- B_CDS _-S_0_, where S_CDS _indicates the signal median of the putative new CDS and B_CDS _indicates the background median of the putative new CDSs. Finally, if S >0, it is regarded as CDS with detectable transcript.

### Reverse transcription (RT) PCR analysis

RT-PCR analysis was conducted using a QIAGEN^®^OneStep RT-PCR Kit following the manufacturer's instructions. The primers used for RT-PCR analysis are listed in Additional file1. Total RNAs were extracted from bacterial culture grown in YEB medium at OD_600 _= 2.0 and a total of 200 ng of total RNA was used for reverse transcription. The cycle number differed in the amplification of different CDS products.

### Generation of in-frame deletion mutants and complementation analysis

Spontaneous rifampicin-resistant derivatives of strain XC1 or 8004 were used as parental strains for generation of deletion mutants. In-frame deletion of *Xcc*_CDS002 (*sir2x*) and *Xcc*_CDS1553 (*arsR*) was conducted using the primers listed in Additional file 1 following the methods described previously [25]. For complementation analysis, the coding regions of *sir2x *and *arsR *respectively were amplified by PCR using the primers listed in Additional file 1 and cloned under the control of *lac *promoter in expression vector pLAFR3. The resultant constructs were transferred into *Xcc *strains through triparental mating.

### Quantitative determination of extracellular enzyme activity, EPS production and virulence test

The extracellular cellulase and protease activity and EPS production in the culture supernatants of *Xcc *strains at OD600 = 2.3 were measured according to the methods described previously [25]. The virulence of *Xcc *to Chinese cabbage was determined following the scissors-clipping method described previously [26]. Fifteen plants were inoculated for each bacterial strain and the experiment was repeated three times.

### Arsenate resistance assay

Sodium arsenate (SIGMA) was added in the following final concentrations (mM): 0.10, 0.25, 0.50, 0.75 and 1.00. Fifty microliters of fresh culture of *Xcc *strain 8004 were inoculated into 5 ml of NYG liquid media with rifampicin (25 μg/ml) and sodium arsenate at different concentrations and grown at 28°C with shaking (250 rpm/min) for overnight. Bacterial growth was indicated by measuring the optical density at 600 nm.

## Competing interests

They authors declare that they have no competing interests.

## Authors' contributions

LZ and FV carried out all the gene prediction, similarity searching. LZ conducted microarray analysis and generated all the mutants. The study was conceived, designed, and coordinated by AP and YWH, who also drafted the manuscript. YQH, BLJ and JLT did the virulence assay. YX was involved in discussion and draft preparation. All authors read and approved the final manuscript.

## Supplementary Material

Additional file 1 **Supplementary tables**. The putative new CDSs identified by similarity searching. The new CDSs identical to the CDSs annotated in *Xcc *strain B100. The new CDSs with detectable transcripts by microarray analysis. Oligos used in this study. Click here for file
