# Supplementary material for: Gene discovery by genome-wide CDS re-prediction and microarray-based transcriptional analysis in phytopathogen Xanthomonas campestris
Source: BMC Genomics. 2011 Jul 12;12:359. doi: 10.1186/1471-2164-12-359 (PMC3142249; doi:10.1186/1471-2164-12-359)
Supplement: Additional file 1 — Supplementary tables. The putative new CDSs identified by similarity searching. The new CDSs identical to the CDSs annotated in Xcc strain B100. The new CDSs with detectable transcripts by microarray analysis. Oligos used in this study. [file 1471-2164-12-359-S1.PDF]

Additional files for Zhou L *et al*:

Gene discovery by genome-wide CDS re-prediction and microarray-based transcriptional analysis in phytopathogen *Xanthomonas campestris*.

Content:

Additional files 1-4

Additional file 1 - The putative new CDSs identified by similarity searching

				Homologues in Nr database		
ID*	Size (aa)	Location in the genome of Xcc 8004 *	Assigned functions	Homologues and putative functions	Size (aa)	E-value
**Xcc_CDS002**	285	5074874 ~ 5075728, (I)	Sir-like regulatory protein	hypothetical protein Shew185_2564 [*Shewanella baltica* OS185]	243	2E-45
				hypothetical protein PSEEN3683 [*Pseudomonas entomophila* L48]	238	3E-45
				hypothetical protein WH5701_01875 [*Synechococcus* sp. WH 5701]	240	2E-37
				hypothetical protein AvinDRAFT_7895 [*Azotobacter vinelandii* AvOP]	294	8E-23
Xcc_CDS005	156	5078626 ~ 5078159, (III)	Hypothetical protein	hypothetical protein xccb100_4410 [Xanthomonas campestris pv. campestris]	54	3E-07
**Xcc_CDS015**	501	5104622 ~ 5106124, (I)	Hypothetical protein	hypothetical protein BURPS1710b_2925 [*Burkholderia pseudomallei* 1710b]	633	7E-52
				conserved hypothetical protein DaciDRAFT_5890 [*Delftia acidovorans* SPH-1]	619	2E-46
				hypothetical protein CtesDRAFT_0331 [*Comamonas testosteroni* KF-1]	527	1E-43
**Xcc_CDS038**	746	5146091 ~ 5148328, (I)	Conserved hypothetical protein	hypothetical protein SmalDRAFT_4031 [*Stenotrophomonas maltophilia* R551-3]	737	1E-130
				conserved hypothetical protein DaciDRAFT_4270 [*Delftia acidovorans* SPH-1]	589	4E-38
				conserved hypothetical protein BmulDRAFT_0150 [*Burkholderia multivorans* ATCC 17616]	557	5E-38
Xcc_CDS095	1011	67926 ~ 70958, (I)	Hemolysin	hemolysin III XOO0165 [*Xanthomonas oryzae* pv. oryzae KACC10331]	1167	0
**Xcc_CDS103**	323	78431 ~ 79399, (I)	Hypothetical protein	hypothetical protein Tery_3666 [Trichodesmium erythraeum IMS101]	251	2E-16
				hypothetical protein MAE_18760 [Microcystis aeruginosa NIES-843]	253	2E-14
				hypothetical protein CY0110_23711 [Cyanothece sp. CCY0110]	249	1E-12
Xcc_CDS105	84	79701 ~ 79450, (III)	Putative secreted protein	putative secreted protein XCV0064 [*Xanthomonas campestris* pv. vesicatoria str. 85-10]	149	1E-23
**Xcc_CDS107**	103	81078 ~ 80770, (III)	Putative secreted protein	putative secreted protein XCV0064 [*Xanthomonas campestris* pv. vesicatoria str. 85-10]	149	5E-50
Xcc_CDS127	134	97042 ~ 97443,	Hypothetical protein	hypothetical protein XC_0297 [Xanthomonas campestris pv. campestris str. 8004]	157	3E-04

		(I)		hypothetical protein XCC0287 [Xanthomonas campestris pv. campestris str. ATCC 33913]	157	3E-04
**Xcc_CDS141**	186	116373 ~ 115816, (III)	Hypothetical protein	hypothetical protein xccb100_0104 [Xanthomonas campestris pv. campestris]	186	1E-101
				hypothetical protein Cpha266_2434 [Chlorobium phaeobacteroides DSM 266]	173	2E-15
				hypothetical protein Ajs_2617 [Acidovorax sp. JS42]	185	2E-11
**Xcc_CDS150**	383	131241 ~ 132389, (II)	Hypothetical protein	hypothetical protein SmalDRAFT_2337 [Stenotrophomonas maltophilia R551-3]	1258	1E-70
				hypothetical protein RAZWK3B_10737 [Roseobacter sp. AzwK-3b]	803	6E-18
				hypothetical protein BURPS1710b_A1230 [Burkholderia pseudomallei 1710b]	827	5E-17
Xcc_CDS247	204	330632 ~ 330021, (III)	Hypothetical protein	hypothetical protein XCC4160 [Xanthomonas campestris pv. campestris str. ATCC 33913]	203	3E-17
				conserved hypothetical protein [Xanthomonas campestris pv. campestris str. 8004]	203	3E-17
**Xcc_CDS251**	303	335769 ~ 334861, (III)	Hypothetical protein	hypothetical protein CC_2256 [Caulobacter crescentus CB15]	379	3E-17
				hypothetical protein sce5319 [Sorangium cellulosum 'So ce 56']	362	9E-16
Xcc_CDS281	110	383359 ~ 383688, (I)	Hypothetical protein	hypothetical protein XAC3497 [Xanthomonas axonopodis pv. citri str. 306]	127	3E-15
				hypothetical protein XAC3417 [Xanthomonas axonopodis pv. citri str. 306]	135	2E-05
Xcc_CDS314	164	412245 ~ 411754, (III)	Hypothetical protein	hypothetical protein Neut_1368 [Nitrosomonas eutropha C91]	165	2E-26
				putative integron gene cassette protein [uncultured bacterium]	154	6E-22
Xcc_CDS318	127	417701 ~ 417321, (IV)	Conserved hypothetical protein	conserved hypothetical protein XOO4230 [Xanthomonas oryzae pv. oryzae KACC10331]	81	8E-23
**Xcc_CDS326**	524	431699 ~ 432750, (I)	Hypothetical protein	hypothetical protein Bpse110_28918 [Burkholderia pseudomallei 1106b]	419	4E-06
				hypothetical protein Bpse17_19685 [Burkholderia pseudomallei 1710a]	419	5E-06
				hypothetical protein BURPS1710b_0247 [Burkholderia pseudomallei 1710b]	423	5E-06
**Xcc_CDS333**	515	441103 ~ 439559, (III)	Hypothetical protein	hypothetical protein BamMEX5DRAFT_6952 [Burkholderia ambifaria MEX-5]	237	3E-23
				hypothetical protein AvinDRAFT_8286 [Azotobacter vinelandii AvOP]	477	8E-19
				hypothetical protein PA2G_01665 [Pseudomonas aeruginosa 2192]	425	3E-12
Xcc_CDS343	379	455643 ~ 454592, (III)	Hypothetical protein	hypothetical protein XOO0229 [Xanthomonas oryzae pv. oryzae KACC10331]	556	4E-82
**Xcc_CDS346**	275	458353 ~ 459177, (I)	Putative cell wall surface anchor family portein	putative cell wall surface anchor family protein BamMC406DRAFT_3465 [Burkholderia ambifaria MC40-6]	313	5E-39
				putative cell wall surface anchor family protein BphytDRAFT_2391 [Burkholderia phytofirmans PsJN]	295	2E-38
				putative cell wall surface anchor family protein RpicDRAFT_1546 [Ralstonia pickettii 12J]	418	4E-38
Xcc_CDS350	592	462280 ~ 463948, (II)	Hypothetical protein	hypothetical protein SmalDRAFT_3802 [Stenotrophomonas maltophilia R551-3]	447	2E-70
				CHAD:Adenylate cyclase CaulDRAFT_5180 [Caulobacter sp. K31]	902	3E-40

**Xcc_CDS356**	142	472327 ~ 471896, (III)	Hypothetical protein	hypothetical protein xccb100_0425 [Xanthomonas campestris pv. campestris]<br>hypothetical protein Xoryp_11220 [Xanthomonas oryzae pv. oryzicola BLS256]<br>hypothetical protein XOO_2682 [Xanthomonas oryzae pv. oryzae MAFF 311018]<br>putative secreted protein [Stenotrophomonas maltophilia R551-3]	162<br>142<br>244<br>131	7E-62<br>1E-08<br>3E-07<br>9E-07
Xcc_CDS360	124	479024 ~ 478653, (III)	Hypothetical protein	unknown protein [Xanthomonas oryzae pv. oryzae KACC10331]<br>unknown protein [Xanthomonas oryzae pv. oryzae KACC10331]	137<br>148	7E-28<br>2E-27
Xcc_CDS372	1276	501670 ~ 499947, (III)	Hypothetical protein	UROPORPHYRINOGEN-III SYNTHASE /Uroporphyrin-III C-methyltransferase [Ralstonia solanacearum UW551]<br>hypothetical protein Adeh_0323 [Anaeromyxobacter dehalogenans 2CP-C]	1065<br>1087	7E-05<br>9E-05
Xcc_CDS389	93	521557 ~ 521279, (III)	Hypothetical protein	hypothetical protein Xoryp_02375 [Xanthomonas oryzae pv. oryzicola BLS256]<br>hypothetical protein XOO_0039 [Xanthomonas oryzae pv. oryzae MAFF 311018]	83<br>83	4E-13<br>3E-12
**Xcc_CDS404**	570	534790 ~ 533033, (III)	Hypothetical protein	Parallel beta-helix repeat protein [Oceanicola granulosus HTCC2516]<br>hypothetical protein BURPS1710b_0494 [Burkholderia pseudomallei 1710b]<br>hypothetical protein BthaB_31371 [Burkholderia thailandensis Bt4]	3143<br>727<br>559	5E-10<br>4E-05<br>1E-04
Xcc_CDS411	153	553624 ~ 553166, (III)	Hypothetical protein	hypothetical protein XCV0499 [Xanthomonas campestris pv. vesicatoria str. 85-10]	248	1E-58
Xcc_CDS424	326	577382 ~ 576405, (III)	Hypothetical protein	Hypothetical protein in CLP 5'region [Xanthomonas campestris (fragment)]<br>hypothetical protein in CLP 5'region [Xanthomonas oryzae pv. oryzae KACC10331]	200<br>278	1E-100<br>2E-89
**Xcc_CDS442**	232	601915 ~ 601196, (III)	Hypothetical protein	hypothetical protein MED121_02085 [Marinomonas sp. MED121]<br>hypothetical protein Daro_2501 [Dechloromonas aromatica RCB]<br>hypothetical protein PST_1635 [Pseudomonas stutzeri A1501]	241<br>235<br>238	8E-17<br>2E-12<br>2E-11
**Xcc_CDS470**	508	655200 ~ 653677, (III)	Hypothetical protein	hypothetical protein SmalDRAFT_3593 [Stenotrophomonas maltophilia R551-3]<br>hypothetical protein RpicDRAFT_1592 [Ralstonia pickettii 12J]<br>conserved hypothetical protein [Burkholderia phytofirmans PsJN]<br>hypothetical protein DaciDRAFT_3105 [Delftia acidovorans SPH-1]	461<br>486<br>414<br>487	6E-88<br>3E-65<br>1E-59<br>4E-59
Xcc_CDS513	144	730945 ~ 730514, (III)	Hypothetical protein	hypothetical protein xccb100_0648 [Xanthomonas campestris pv. campestris]	103	6E-19
Xcc_CDS523	41	743828 ~ 743706, (III)	Hypothetical protein	hypothetical protein PXO_05814 [Xanthomonas oryzae pv. oryzae PXO99A]	41	3E-11
Xcc_CDS525	65	745807 ~ 745613, (III)	Hypothetical protein	hypothetical protein PXO_04175 [Xanthomonas oryzae pv. oryzae PXO99A]	38	2E-12

Xcc_CDS528	257	752771 ~ 753541, (I)	Endonuclease	endonuclease XOO4028 [Xanthomonas oryzae pv. oryzae KACC10331]	352	2E-32
**Xcc_CDS538**	801	763903 ~ 766305, (I)	Hypothetical protein	hypothetical protein SmalDRAFT_1790 [Stenotrophomonas maltophilia R551-3]	787	1E-88
				conserved hypothetical protein [Comamonas testosteroni KF-1]	616	3E-28
				hypothetical protein Rpic12DDRAFT_3212 [Ralstonia pickettii 12D]	529	2E-25
Xcc_CDS575	754	835331 ~ 833070, (III)	Hypothetical protein	hypothetical protein BamIOP4010DRAFT_6698 [Burkholderia ambifaria IOP40-10]	697	5E-20
				hypothetical protein BamMEX5DRAFT_6912 [Burkholderia ambifaria MEX-5]	460	2E-16
**Xcc_CDS594**	523	902129 ~ 900465, (III)	Hypothetical protein	hypothetical protein XOO3916 [Xanthomonas oryzae pv. oryzae KACC10331]	375	1E-113
				hypothetical protein CaulDRAFT_3196 [Caulobacter sp. K31]	404	8E-41
Xcc_CDS604	286	926807 ~ 927664, (I)	Hypothetical protein	hypothetical protein BpseN_08019 [Burkholderia pseudomallei NCTC 13177]	579	5E-13
				hypothetical protein BURPS1710b_1624 [Burkholderia pseudomallei 1710b]	579	5E-13
Xcc_CDS639	806	984755 ~ 982338, (III)	Chloramphenicol O-acetyltransferase	Chloramphenicol O-acetyltransferase [Methylobacterium chloromethanicum CM4]	1060	1E-102
**Xcc_CDS658**	754	1021828 ~ 1019567, (III)	Conserved hypothetical protein	conserved hypothetical protein SmalDRAFT_3283 [Stenotrophomonas maltophilia R551-3]	602	1E-178
				conserved hypothetical protein [Burkholderia ambifaria MC40-6]	760	4E-125
				hypothetical protein Rpic12DDRAFT_4684 [Ralstonia pickettii 12D]	1005	9E-121
				conserved hypothetical protein [Delftia acidovorans SPH-1]	762	3E-103
Xcc_CDS693	250	1094613 ~ 1093852, (III)	Hypothetical protein	conserved hypothetical protein [Rhizobium leguminosarum bv. trifolii WSM1325]	287	4E-07
				hypothetical protein BuboB_13967 [Burkholderia ubonensis Bu]	343	1E-05
Xcc_CDS702	125	1110793 ~ 1110419, (III)	Hypothetical protein	unknown protein [Xanthomonas oryzae pv. oryzae KACC10331]	398	4E-28
				unknown protein [Xanthomonas oryzae pv. oryzae KACC10331]	137	9E-28
Xcc_CDS708	132	1117050 ~ 1117445, (I)	Hypothetical protein	hypothetical protein XOO0408 [Xanthomonas oryzae pv. oryzae KACC10331]	129	4E-06
				hypothetical protein XAC3497 [Xanthomonas axonopodis pv. citri str. 306]	127	6E-04
**Xcc_CDS740**	547	1205358 ~ 1203718, (III)	Conserved hypothetical protein	conserved hypothetical protein SmalDRAFT_3164 [Stenotrophomonas maltophilia R551-3]	551	1E-104
				conserved hypothetical protein BamMC406DRAFT_1213 [Burkholderia ambifaria MC40-6]	527	1E-16
				hypothetical protein BURPS1710b_3714 [Burkholderia pseudomallei 1710b]	495	1E-16
**Xcc_CDS771**	122	1254035 ~ 1254400, (I)	Conserved hypothetical protein	conserved hypothetical protein DaciDRAFT_5957 [Delftia acidovorans SPH-1]	119	7E-25
				hypothetical protein XCV2359 [Xanthomonas campestris pv. vesicatoria str. 85-10]	120	2E-23
				hypothetical protein Ajs_1390 [Acidovorax sp. JS42]	120	2E-23
				conserved hypothetical protein CtesDRAFT_3430 [Comamonas testosteroni KF-1]	119	3E-23
				hypothetical protein Paer2_01002189 [Pseudomonas aeruginosa 2192]	119	2E-14

**Xcc_CDS801**	489	1296479 ~ 1297945, (I)	Hypothetical protein	hypothetical protein BAL199_18821 [alpha proteobacterium BAL199]	486	2E-38
				hypothetical protein BoklE_05515 [Burkholderia oklahomensis EO147]	559	1E-26
				hypothetical protein BoklC_05370 [Burkholderia oklahomensis C6786]	559	2E-26
**Xcc_CDS834**	600	1372065 ~ 1370266, (III)	Hypothetical protein	hypothetical protein Bpse17_10908 [Burkholderia pseudomallei 1710a]	595	1E-101
				hypothetical protein Bpse14_03871 [Burkholderia pseudomallei 14]	595	1E-101
				hypothetical protein Bpseu9_03866 [Burkholderia pseudomallei 9]	595	1E-101
Xcc_CDS837	203	1375206 ~ 1374598, (III)	Conserved hypothetical protein	conserved hypothetical protein [Ralstonia pickettii 12D]	214	3E-12
**Xcc_CDS839**	884	1378682 ~ 1376031, (III)	Conserved hypothetical protein	conserved hypothetical protein SmalDRAFT_3094 [Stenotrophomonas maltophilia R551-3]	687	0
				hypothetical protein BURPS1710b_2340 [Burkholderia pseudomallei 1710b]	738	2E-79
				conserved hypothetical protein M446DRAFT_5163 [Methylobacterium sp. 4-46]	668	8E-67
**Xcc_CDS842**	254	1385415 ~ 1384654, (III)	Hypothetical protein	hypothetical protein Sententerica_06636 [Salmonella enterica subsp. enterica serovar Kentucky str. CDC 191]	218	1E-06
				hypothetical protein ESA_02602 [Enterobacter sakazakii ATCC BAA-894]	216	3E-04
				hypothetical protein EschericoliO157_02910 [Escherichia coli O157:H7 str. EC4045]	206	4E-04
Xcc_CDS846	231	1387605 ~ 1386913, (III)	Hypothetical protein	hypothetical protein SKA58_05360 [Sphingomonas sp. SKA58]	447	2E-15
				hypothetical protein MchlDRAFT_5428 [Methylobacterium chloromethanicum CM4]	258	9E-05
				hypothetical protein BmalP_25215 [Burkholderia mallei PRL-20]	497	8E-04
Xcc_CDS900	170	1474504 ~ 1474844, (II)	Outer protein	Xanthomonas outer protein D XCV0437 [Xanthomonas campestris pv. vesicatoria str. 85-10]	545	4E-49
**Xcc_CDS866**	229	1403462 ~ 1404148, (I)	Hypothetical protein	hypothetical protein Bpse7_18771 [Burkholderia pseudomallei 7894]	624	2E-14
				hypothetical protein BpseB_18162 [Burkholderia pseudomallei B7210]	565	2E-14
				hypothetical protein Bpse9_19369 [Burkholderia pseudomallei 91]	586	2E-14
Xcc_CDS924	153	1507552 ~ 1508000, (I)	Hypothetical protein	hypothetical protein XAC3059 [Xanthomonas axonopodis pv. citri str. 306]	199	2E-36
				hypothetical protein XOO1796 [Xanthomonas oryzae pv. oryzae KACC10331]	360	2E-35
**Xcc_CDS936**	605	1528313 ~ 1530127, (I)	Hypothetical protein	hypothetical protein EcE24377A_4969 [Escherichia coli E24377A]	496	8E-45
				conserved hypothetical protein [Stenotrophomonas maltophilia R551-3]	747	3E-11
				hypothetical protein Xoryp_05010 [Xanthomonas oryzae pv. oryzicola BLS256]	738	1E-06
**Xcc_CDS947**	436	1554197 ~ 1555504, (I)	Conserved hypothetical protein	conserved hypothetical protein SmalDRAFT_2998 [Stenotrophomonas maltophilia R551-3]	442	1E-88
				hypothetical protein BURPS1710b_0171 [Burkholderia pseudomallei 1710b]	465	1E-41
				conserved hypothetical protein [Burkholderia cenocepacia MC0-3]	403	1E-38
Xcc_CDS1002	253	1645689 ~ 1644931, (III)	Hypothetical protein	unknown protein XOO1421 [Xanthomonas oryzae pv. oryzae KACC10331]	247	9E-47
				hypothetical protein CC_1050 [Caulobacter crescentus CB15]	265	5E-06

**Xcc_CDS1047**	278	1715471 ~ 1714638, (III)	Hypothetical protein	hypothetical protein BpseN_08019 [Burkholderia pseudomallei NCTC 13177]	579	3E-08
				hypothetical protein BURPS1710b_1624[Burkholderia pseudomallei 1710b]	579	3E-08
				hypothetical protein Bpse14_08382 [Burkholderia pseudomallei 14]	579	1E-07
Xcc_CDS1060	174	1736932 ~ 1736411, (III)	Hypothetical protein	transcriptional regulator XOO1507 [Xanthomonas oryzae pv. oryzae KACC10331]	477	3E-28
				hypothetical protein XF1767 [Xylella fastidiosa 9a5c]	356	8E-12
**Xcc_CDS1079**	638	1775688 ~ 1773775, (III)	Hypothetical protein	hypothetical protein BamMEX5DRAFT_6750 [Burkholderia ambifaria MEX-5]	472	1E-13
				Hypothetical protein COLAER_01410 [Collinsella aerofaciens ATCC 25986]	772	3E-12
Xcc_CDS1091	56	1797312 ~ 1797145, (III)	Hypothetical protein	hypothetical protein Xoryp_08065 [Xanthomonas oryzae pv. oryzicola BLS256]	58	1E-04
**Xcc_CDS1095**	1283	1804264 ~1800416, (III)	Conserved hypothetical protein	conserved hypothetical protein PputGB1DRAFT_1122 [Pseudomonas putida GB-1]	840	1E-121
				hypothetical protein RpicDRAFT_1602 [Ralstonia pickettii 12J]	1094	1E-109
				conserved hypothetical protein Bcenmc03DRAFT_3464 [Burkholderia cenocepacia MC0-3]	903	1E-103
**Xcc_CDS1100**	537	1823106 ~ 1821496, (III)	Hypothetical protein	hypothetical protein Bpse9_19204 [Burkholderia pseudomallei 91]	537	2E-05
				hypothetical protein Bpse112_18109 [Burkholderia pseudomallei 112]	537	4E-05
				hypothetical protein BpseN_17991 [Burkholderia pseudomallei NCTC 13177]	537	4E-05
Xcc_CDS1110	168	1844742 ~ 1844239, (III)	Hypothetical protein	NLP/P60 [Stenotrophomonas maltophilia R551-3]	376	5E-40
				hypothetical protein ESA_04392 [Enterobacter sakazakii ATCC BAA-894]	184	3E-06
**Xcc_CDS1129**	415	1885910 ~ 1884642, (III)	Conserved hypothetical protein	conserved hypothetical protein SmalDRAFT_2817 [Stenotrophomonas maltophilia R551-3]	484	1E-111
				conserved hypothetical protein PputGB1DRAFT_4592 [Pseudomonas putida GB-1]	392	2E-62
				conserved hypothetical protein [Burkholderia multivorans ATCC 17616]	424	2E-57
Xcc_CDS1134	447	1895218 ~ 1893878, (III)	Hypothetical protein	hypothetical protein BACUNI_01874 [Bacteroides uniformis ATCC 8492]	248	3E-21
Xcc_CDS1137	463	1898968 ~ 1897532, (III)	Hypothetical protein	hypothetical protein AchlDRAFT_3524 [Arthrobacter chlorophenolicus A6]	248	2E-10
Xcc_CDS1143	170	1911970 ~1911461, (III)	Hypothetical protein	hypothetical protein XOO3231 [Xanthomonas oryzae pv. oryzae KACC10331]	191	3E-73
**Xcc_CDS1147**	784	1922714 ~ 1920363, (III)	Hypothetical protein	hypothetical protein SmalDRAFT_2789 [Stenotrophomonas maltophilia R551-3]	722	1E-125
				hypothetical protein Bcenmc03DRAFT_5785 [Burkholderia cenocepacia MC0-3]	989	1E-118
				hypothetical protein DaciDRAFT_5850 [Delftia acidovorans SPH-1]	910	1E-109
**Xcc_CDS1152**	767	1932962 ~ 1930662, (III)	Conserved hypothetical protein	conserved hypothetical SmalDRAFT_2787 [Stenotrophomonas maltophilia R551-3]	798	0
				conserved hypothetical protein RpicDRAFT_1519 [Ralstonia pickettii 12J]	752	1E-121
				conserved hypothetical protein Bcenmc03DRAFT_5786 [Burkholderia cenocepacia MC0-3]	822	e-113
Xcc_CDS1182	429	1989793 ~ 1988507,	Hypothetical protein	hypothetical protein Bpse110_12948 [Burkholderia pseudomallei 1106b]	1039	9E-31

		(III)		hypothetical protein BURPS1710b_1895 [Burkholderia pseudomallei 1710b]	1039	2E-30
**Xcc_CDS1183**	797	1992481 ~ 1990091,	Hypothetical protein	hypothetical protein Bpse110_12948 [Burkholderia pseudomallei 1106b]	1039	4E-52
		(III)		hypothetical protein BURPS1710b_1895 [Burkholderia pseudomallei 1710b]	1039	9E-52
				hypothetical protein Rpic12DDRAFT_0304 [Ralstonia pickettii 12D]	853	2E-51
				hypothetical protein CtesDRAFT_1056 [Comamonas testosteroni KF-1]	870	3E-50
Xcc_CDS1196	140	2032607 ~ 2033026,	Hypothetical protein	hypothetical protein BthaT_24240 [Burkholderia thailandensis TXDOH]	108	8E-05
		(I)		hypothetical protein ebA1085 [Azoarcus sp. EbN1]	107	1E-04
				hypothetical protein Bpse38_12048 [Burkholderia thailandensis MSMB43]	107	4E-04
Xcc_CDS1219	670	2086440 ~ 2084602,	Hypothetical protein	hypothetical protein BthaT_06351 [Burkholderia thailandensis TXDOH]	524	4E-13
		(III)		hypothetical protein BmalP_28255 [Burkholderia mallei PRL-20]	524	3E-12
				hypothetical protein BpseBC_30682 [Burkholderia pseudomallei BCC215]	524	3E-12
**Xcc_CDS1224**	384	2098936 ~2097785,	Conserved hypothetical protein	conserved hypothetical protein Bcenmc03DRAFT_3605 [Burkholderia cenocepacia MC0-3]	431	7E-35
		(III)		conserved hypothetical protein BmulDRAFT_3895 [Burkholderia multivorans ATCC 17616]	406	5E-31
				hypothetical protein DaciDRAFT_3150 [Delftia acidovorans SPH-1]	467	4E-30
Xcc_CDS1267	84	2193987 ~ 2194238,	Hypothetical protein	hypothetical protein XAC2559 [Xanthomonas axonopodis pv. citri str. 306]	91	3E-07
		((II)		hypothetical protein XCV2759 [Xanthomonas campestris pv. vesicatoria str. 85-10]	108	3E-07
				hypothetical protein XCC2311 [Xanthomonas campestris pv. campestris str. ATCC 33913]	116	3E-07
				hypothetical protein Xoryp_09180 [Xanthomonas oryzae pv. oryzicola BLS256]	140	4E-07
**Xcc_CDS1274**	495	2219040 ~ 2220524,	Hypothetical protein	hypothetical protein ESA_04230 [Enterobacter sakazakii ATCC BAA-894]	375	8E-32
		(IV)		hypothetical protein Bpse110_09294 [Burkholderia pseudomallei 1106b]	735	4E-14
				hypothetical protein BURPS1710b_A0838 [Burkholderia pseudomallei 1710b]	745	4E-14
Xcc_CDS1280	247	2233392 ~ 2232652,	Hypothetical protein	hypothetical protein Bpseu9_41232 [Burkholderia pseudomallei 9]	753	4E-09
		(III)		hypothetical protein BURPS1710b_2687 [Burkholderia pseudomallei 1710b]	755	5E-09
Xcc_CDS1281	281	2238855 ~ 2238013,	Hypothetical protein	hypothetical protein CKO_03686 [Citrobacter koseri ATCC BAA-895]	235	8E-16
		(III)		hypothetical protein c5233 [Escherichia coli CFT073]	184	2E-13
				hypothetical protein EcolO_26931 [Escherichia coli O157:H7 str. EC508]	184	8E-13
Xcc_CDS1297	57	2262146 ~ 2262316,	Hypothetical protein	hypothetical protein PXO_05567 [Xanthomonas oryzae pv. oryzae PXO99A]	57	7E-09
		(I)				
Xcc_CDS1302	546	2275261~ 2274418,	Conserved hypothetical protein	conserved hypothetical protein [Pseudomonas putida GB-1]	467	4E-42
		(III)		hypothetical protein PmenDRAFT_2946 [Pseudomonas mendocina ymp]		3E-39
**Xcc_CDS1309**	424	2294965 ~ 2294112,	ABC transporter heme permease	ABC transporter heme permease [Xanthomonas oryzae pv. oryzae KACC10331]	955	1E-127
		(III)				
Xcc_CDS1317	438	2305518 ~ 2304205,	Conserved hypothetical protein	conserved hypothetical protein [Xanthomonas oryzae pv. oryzae KACC10331]	390	1E-41
		(III)				

Xcc_CDS1323	225	2323020 ~ 2322346, (III)	Hypothetical protein	hypothetical protein BpseBC_15624 [Burkholderia pseudomallei BCC215]	802	9E-21
				hypothetical protein BpseN_15892 [Burkholderia pseudomallei NCTC 13177]	803	9E-21
				hypothetical protein Bpse110_14044 [Burkholderia pseudomallei 1106b]	802	9E-21
**Xcc_CDS1324**	601	2324852 ~ 2323050, (III)	Conserved hypothetical protein	conserved hypothetical protein PputW619DRAFT_1953 [Pseudomonas putida W619]	595	1E-123
				conserved hypothetical protein DaciDRAFT_5891 [Delftia acidovorans SPH-1]	674	1E-111
				conserved hypothetical protein BamMC406DRAFT_1774 [Burkholderia ambifaria MC40-6]	554	1E-108
				conserved hypothetical protein CtesDRAFT_0332 [Comamonas testosteroni KF-1]	614	1E-102
				hypothetical protein MchlDRAFT_1316 [Methylobacterium chloromethanicum CM4]	602	5E-75
**Xcc_CDS1340**	502	2351256 ~ 2352761, (I)	Hypothetical protein	hypothetical protein RlegDRAFT_6079 [Rhizobium leguminosarum bv. trifolii WSM1325]	246	1E-36
				hypothetical protein Bpse110_32381 [Burkholderia pseudomallei 1106b]	587	4E-05
				hypothetical protein BURPS1710b_0494 [Burkholderia pseudomallei 1710b]	727	1E-04
Xcc_CDS1342	308	2353815 ~ 2352892, (III)	Putative GTPase	predicted GTPases [Xanthomonas oryzae pv. oryzae KACC10331]	487	4E-77
Xcc_CDS1353	84	2373980 ~ 2374231, (I)	Hypothetical protein	hypothetical protein xccb100_2031 [Xanthomonas campestris pv. campestris]	88	3E-09
				hypothetical protein S2640 [Shigella flexneri 2a str. 2457T]	111	1E-06
				hypothetical protein XCV2226 [Xanthomonas campestris pv. vesicatoria str. 85-10]	66	0.001
Xcc_CDS1361	693	2396844 ~ 2394700, (III)	Hypothetical protein	hypothetical protein BthaT_03759 [Burkholderia thailandensis TXDOH]	553	1E-51
				hypothetical protein BthaB_03789 [Burkholderia thailandensis Bt4]	553	2E-51
Xcc_CDS1376	311	2425207 ~ 2424275, (III)	Hypothetical protein	unknown protein XOO0021 [Xanthomonas oryzae pv. oryzae KACC10331]	316	1E-101
				unknown protein XOO3091 [Xanthomonas oryzae pv. oryzae KACC10331]	213	2E-60
**Xcc_CDS1381**	181	2428687 ~ 2428145, (III)	Putative secreted protein	putative secreted protein Aave_0536 [Acidovorax avenae subsp. citrulli AAC00-1]	142	2E-21
				putative secreted protein XCV1811 [Xanthomonas campestris pv. vesicatoria str. 85-10]	144	2E-15
				hypothetical protein XAC1779 [Xanthomonas axonopodis pv. citri str. 306	155	4E-12
**Xcc_CDS1401**	283	2470019 ~ 2469171, (IV)	Conserved hypothetical protein	conserved hypothetical protein [Parvibaculum lavamentivorans DS-1]	270	5E-61
				hypothetical protein Bxe_A1215 [Burkholderia xenovorans LB400]	270	1E-60
				hypothetical protein XCV2398 [Xanthomonas campestris pv. vesicatoria str. 85-10]	270	3E-60
				conserved hypothetical protein DaciDRAFT_5988 [Delftia acidovorans SPH-1]	284	5E-59
				conserved hypothetical protein CtesDRAFT_3384 [Comamonas testosteroni KF-1]	274	1E-58
				hypothetical protein Rmet_2311 [Ralstonia metallidurans CH34]	274	1E-58
				hypothetical protein PA2G_02094 [Pseudomonas aeruginosa 2192]	274	1E-58
Xcc_CDS1404	61	2474467 ~ 2474285, (IV) Xcc strain 8004-specific	Hypothetical protein	hypothetical protein XCV2285 [Xanthomonas campestris pv. vesicatoria str. 85-10]	70	4E-27
				hypothetical protein Bxe_A1220 [Burkholderia xenovorans LB400]	128	9E-24
				hypothetical protein XF1772 [Xylella fastidiosa 9a5c]	136	9E-24
				hypothetical protein ORF88400 [Pseudomonas putida]	70	9E-24
				conserved hypothetical protein DaciDRAFT_5992 [Delftia acidovorans SPH-1]	70	1E-22
				conserved hypothetical protein CtesDRAFT_3379 [Comamonas testosteroni KF-1]	73	1E-22
				hypothetical protein PaerPA_01003096 [Pseudomonas aeruginosa PACS2]	70	2E-22
				hypothetical protein Rmet_2306 [Ralstonia metallidurans CH34]	137	4E-22

**Xcc_CDS1416**	533	2489339 ~ 2490937, (II)	DNA methylase	putative DNA methylase [Verminephrobacter eiseniae EF01-2]	928	0
				putative DNA methylase [Roseovarius sp. 217]	920	0
				putative DNA methylase [Erythrobacter sp. NAP1]	909	0
				putative DNA methylase [Acidovorax sp. JS42]	928	0
**Xcc_CDS1436**	1272	2521043 ~ 2524858, (I)	Hypothetical protein	hypothetical protein BgramDRAFT_5354 [Burkholderia graminis C4D1M]	872	2E-89
				hypothetical protein MchlDRAFT_2828 [Methylobacterium chloromethanicum CM4]	996	3E-36
				hypothetical protein RlegDRAFT_3989 [Rhizobium leguminosarum bv. trifolii WSM1325]	673	4E-35
Xcc_CDS1446	102	2536984 ~ 2536679, (III)	Transmembrane protein	hypothetical protein XOO4747 [Xanthomonas oryzae pv. oryzae KACC10331]	64	4E-13
				Chloride channel protein EriC [Xanthomonas oryzae pv. oryzae PXO99A]	64	4E-13
				probable transmembrane protein [Xanthomonas oryzae pv. oryzae PXO99A]	64	2E-12
Xcc_CDS1461	329	2565755 ~ 2566741, (II)	Hypothetical protein	conserved hypothetical protein SmalDRAFT_1971 [Stenotrophomonas maltophilia R551-3]	92	2E-33
				hypothetical protein XAC2135 [Xanthomonas axonopodis pv. citri str. 306]	92	2E-30
				hypothetical protein XCV2117 [Xanthomonas campestris pv. vesicatoria str. 85-10]	74	2E-27
Xcc_CDS1496	154	2611551 ~ 2611090, (III)	Hypothetical protein	hypothetical protein XOO3088 [Xanthomonas oryzae pv. oryzae KACC10331]	115	2E-19
				hypothetical protein XAC2156 [Xanthomonas axonopodis pv. citri str. 306]	85	1E-17
				hypothetical protein XCV2097 [Xanthomonas campestris pv. vesicatoria str. 85-10]	85	1E-17
				conserved hypothetical protein SmalDRAFT_3506 [Stenotrophomonas maltophilia R551-3]	99	6E-06
				hypothetical protein alr1718 [Nostoc sp. PCC 7120]	109	8E-06
				hypothetical protein Ava_0312 [Anabaena variabilis ATCC 29413]	122	1E-05
**Xcc_CDS1499**	986	2622928 ~ 2625885, (I)	Conserved hypothetical protein	hypothetical protein BURPS1710b_0742 [Burkholderia pseudomallei 1710b]	953	1E-156
				conserved hypothetical protein DaciDRAFT_2072 [Delftia acidovorans SPH-1]	455	2E-73
				conserved hypothetical protein RpicDRAFT_2851 [Ralstonia pickettii 12J]	421	4E-73
Xcc_CDS1508	132	2651747 ~ 2652142, (I)	Hypothetical protein	hypothetical protein Bpse38_07861 [Burkholderia thailandensis MSMB43]	129	6E-19
				hypothetical protein BuboB_17007 [Burkholderia ubonensis Bu]	129	4E-13
				hypothetical protein CKO_00266 [Citrobacter koseri ATCC BAA-895]	137	2E-10
				hypothetical protein RCCS2_01269 [Roseobacter sp. CCS2]	155	2E-08
Xcc_CDS1552	59	2759245 ~ 2759069, (IV)	Hypothetical protein	hypothetical protein XOO2822 [Xanthomonas oryzae pv. oryzae KACC10331]	148	4E-17
				hypothetical protein XOO_2675 [Xanthomonas oryzae pv. oryzae MAFF 311018]	83	4E-17
				hypothetical protein XCV1970 [Xanthomonas campestris pv.vesicatoria str. 85-10]	148	1E-14
**Xcc_CDS1553**	116	2764864 ~ 2764517, (IV)	Regulatory protein ArsR	regulatory protein ArsR Oant_3211 [Ochrobactrum anthropi ATCC 49188]	121	4E-34
				regulatory protein, ArsR RPD_3639 [Rhodopseudomonas palustris BisB5]	130	1E-29
				regulatory protein ArsR A1S_0654 [Acinetobacter baumannii ATCC 17978]	91	1E-22
				regulatory protein ArsR [Xanthobacter autotrophicus Py2]	119	2E-18
				Transcriptional regulatory protein Nwi_3123[Nitrobacter winogradskyi Nb-255]	120	9E-18
				regulatory protein PdenDRAFT_3911, ArsR [Paracoccus denitrificans PD1222]	120	2E-16
				regulatory protein MchlDRAFT_0423, ArsR [Methylobacterium chloromethanicum CM4]	119	3E-16
Xcc_CDS1559	108	2769250 ~ 2768927,	Hypothetical protein	hypothetical protein xccb100_2180 [Xanthomonas campestris pv. campestris]	103	3E-27

		(III)				
**Xcc_CDS1561**	689	2775992 ~ 2774779, (III)	Hypothetical protein	conserved hypothetical protein [Ralstonia pickettii 12D]	837	2E-55
				hypothetical protein Bpse110_29933 [Burkholderia pseudomallei 1106b]	1004	5E-46
				hypothetical protein Bpse17_20680 [Burkholderia pseudomallei 1710a]	1007	3E-45
**Xcc_CDS1569**	537	2787422 ~ 2785811, (III)	Conserved hypothetical protein	hypothetical protein SmalDRAFT_1826 [Stenotrophomonas maltophilia R551-3]	753	9E-61
				conserved hypothetical protein [Comamonas testosteroni KF-1]	616	7E-48
				conserved hypothetical protein [Ralstonia pickettii 12D]	575	7E-40
Xcc_CDS1577	1188	2800075 ~ 2796512, (III)	Hypothetical protein	hypothetical protein SmalDRAFT_1826 [Stenotrophomonas maltophilia R551-3]	753	2E-64
**Xcc_CDS1585**	531	2824681~ 2826273, (I)	Conserved hypothetical protein	conserved hypothetical protein SmalDRAFT_1787 [Stenotrophomonas maltophilia R551-3]	574	1E-146
				conserved hypothetical protein BamMC406DRAFT_2943 [Burkholderia ambifaria MC40-6]	533	2E-73
				conserved hypothetical protein Bcenmc03DRAFT_5197 [Burkholderia cenocepacia MC0-3]	532	8E-71
				conserved hypothetical protein AvinDRAFT_3382 [Azotobacter vinelandii AvOP]	524	3E-70
				conserved hypothetical protein PputW619DRAFT_2673 [Pseudomonas putida W619]	565	2E-67
				hypothetical protein DaciDRAFT_5844 [Delftia acidovorans SPH-1]	797	8E-63
				conserved hypothetical protein CtesDRAFT_5141 [Comamonas testosteroni KF-1]	504	3E-46
Xcc_CDS1586	429	2826176 ~ 2827462, (IV)	Hypothetical protein	hypothetical protein PputGB1DRAFT_4893 [Pseudomonas putida GB-1]	924	2E-72
				hypothetical protein BmulDRAFT_2372 [Burkholderia multivorans ATCC 17616]	710	5E-60
**Xcc_CDS1588**	427	2842397 ~ 2843677, (I)	Conserved hypothetical protein	hypothetical protein BmulDRAFT_2094 [Burkholderia multivorans ATCC 17616]	542	7E-65
				conserved hypothetical protein BphytDRAFT_2573 [Burkholderia phytofirmans PsJN]	438	2E-61
				conserved hypothetical protein Bcenmc03DRAFT_5793 [Burkholderia cenocepacia MC0-3]	444	3E-58
				conserved hypothetical protein BamMC406DRAFT_6047 [Burkholderia ambifaria MC40-6]	397	2E-21
Xcc_CDS1596	515	2852977 ~ 2854521, (I)	Hypothetical protein	hypothetical protein BpseN_12074 [Burkholderia pseudomallei NCTC 13177]	847	2E-09
				hypothetical protein BmalP_23380 [Burkholderia mallei PRL-20]	847	3E-09
				hypothetical protein MchlDRAFT_3166 [Methylobacterium chloromethanicum CM4]	611	1E-07
**Xcc_CDS1604**	282	2867174 ~ 2868019, (I)	Hypothetical protein	conserved hypothetical protein [Pseudomonas aeruginosa 2192]	278	8E-24
				hypothetical protein SYNPCC7002_A1635 [Synechococcus sp. PCC 7002]	249	2E-06
Xcc_CDS1617	91	2884161 ~ 2883890, (III)	tryptophan-rich sensory protein	putative tryptophan-rich sensory protein [Xanthomonas campestris pv. vesicatoria str. 85-10]	166	2E-06
				tryptophan-rich sensory protein [Xanthomonas axonopodis pv. citri str. 306]	166	4E-04
**Xcc_CDS1619**	245	2886681 ~ 2885857, (III)	Hypothetical protein	PUTATIVE TRANSMEMBRANE PROTEIN RSp1070 [Ralstonia solanacearum GMI1000]	305	7E-45
				hypothetical protein PFL_2345 [Pseudomonas fluorescens Pf-5]	311	9E-09
				hypothetical protein AHA_1829 [Aeromonas hydrophila subsp. hydrophila ATCC 7966]	329	1E-08
				conserved hypothetical protein A33_A0409 [Vibrio cholerae AM-19226]	321	3E-08
**Xcc_CDS1633**	117	2926523 ~ 2926173, (III)	Transcriptional regulators	transcriptional regulator XCV1100 [Xanthomonas campestris pv. vesicatoria str. 85-10]	118	3E-05
				Predicted transcriptional regulators YmolA_01000705 [Yersinia mollaretii ATCC 43969]	109	7E-05
				putative transcriptional regulator P3TCK_08653 [Photobacterium profundum 3TCK]	102	2E-04

				transcriptional regulator XAC1499 [Xanthomonas axonopodis pv. citri str. 306]	98	2E-04
				transcriptional regulator Sbal223DRAFT_4100 [Shewanella baltica OS223]	104	3E-04
Xcc_CDS1646	96	2933205 ~ 2932918, (III)	Hypothetical protein	hypothetical protein SO_A0059 [Shewanella oneidensis MR-1]	96	3E-10
				Uncharacterized protein Haso02000551 conserved in bacteria [Haemophilus somnus 2336]	99	1E-08
				hypothetical protein SSON_0410 [Shigella sonnei Ss046]	119	2E-08
				Uncharacterized protein conserved in bacteria [Escherichia coli E24377A]	103	2E-08
				hypothetical protein BBta_4869 [Bradyrhizobium sp. BTAi1]	100	3E-07
Xcc_CDS1689	298	3023669 ~ 3022776, (IV)	dihydroxydipicolinate synthase	dihydroxydipicolinate synthase XOO2923 [Xanthomonas oryzae pv. oryzae KACC10331]	545	2E-30
**Xcc_CDS1718**	409	3061241 ~ 3062467, (I)	Conserved hypothetical protein	conserved hypothetical protein SmalDRAFT_1402 [Stenotrophomonas maltophilia R551-3]	531	1E-118
				conserved hypothetical protein AvinDRAFT_3338 [Azotobacter vinelandii AvOP]	447	9E-66
				hypothetical protein Rpic12DDRAFT_2472 [Ralstonia pickettii 12D]	629	3E-63
				conserved hypothetical protein PputW619DRAFT_2672 [Pseudomonas putida W619]	448	3E-55
				hypothetical protein MchlDRAFT_3857 [Methylobacterium chloromethanicum CM4]	415	2E-54
				hypothetical protein BmulDRAFT_2376 [Burkholderia multivorans ATCC 17616]	402	8E-54
**Xcc_CDS1719**	298	3062814 ~ 3063707, (I)	Hypothetical protein	hypothetical protein XOO2965 [Xanthomonas oryzae pv. oryzae KACC10331]	317	1E-113
				hypothetical protein PputW619DRAFT_1957 [Pseudomonas putida W619]	589	3E-24
				hypothetical protein Anae109_4317 [Anaeromyxobacter sp. Fw109-5]	241	3E-21
Xcc_CDS1720	591	3063918 ~ 3065690, (I)	Hypothetical protein	hypothetical protein ESA_00522 [Enterobacter sakazakii ATCC BAA-894]	538	7E-70
				Hypothetical protein COLAER_00348 [Collinsella aerofaciens ATCC 25986]	439	4E-26
**Xcc_CDS1746**	134	3122564 ~ 3122037, (III)	Hypothetical protein	hypothetical protein Pfl_2792 [Pseudomonas fluorescens PfO-1]	106	3E-20
				hypothetical protein XCV3887 [Xanthomonas campestris pv. vesicatoria str. 85-10]	123	2E-17
				hypothetical protein NAP1_11268 [Erythrobacter sp. NAP1]	120	3E-17
				hypothetical protein PSPPH_3474 [Pseudomonas syringae pv. Phaseolicola 1448A]	118	1E-16
				hypothetical protein ELI_10905 [Erythrobacter litoralis HTCC2594]	106	7E-16
				hypothetical protein BBta_3323 [Bradyrhizobium sp. BTAi1]	108	2E-14
				hypothetical protein XAC3775 [Xanthomonas axonopodis pv. citri str. 306]	89	2E-13
				hypothetical protein Krad_0270 [Kineococcus radiotolerans SRS30216]	128	6E-13
Xcc_CDS1751	178	3131707 ~ 3131174, (I)	Unknown protein	unknown protein [Xanthomonas oryzae pv. oryzae KACC10331]	398	2E-27
				unknown protein [Xanthomonas oryzae pv. oryzae KACC10331]	137	5E-27
**Xcc_CDS1757**	141	3140508 ~ 3140952, (I)	Hypothetical protein	hypothetical protein XACb0003 [Xanthomonas axonopodis pv. citri str. 306]	237	7E-56
Xcc_CDS1761	97	3148900 ~ 3149190, (I)	Hypothetical protein	hypothetical protein ECA0275 [Erwinia carotovora subsp. atroseptica SCRI1043]	112	1E-17
				hypothetical protein PFL_1370 [Pseudomonas fluorescens Pf-5]	120	2E-17
				hypothetical protein Mmwyl1_1177 [Marinomonas sp. MWYL1]	101	2E-16
				conserved hypothetical protein Sput200DRAFT_1692 [Shewanella putrefaciens 200]	98	2E-15
**Xcc_CDS1770**	285	3164786 ~ 3163932, (III)	Hypothetical protein	hypothetical protein Xoryp_18600 [Xanthomonas oryzae pv. oryzicola BLS256]	306	5E-97
				conserved hypothetical protein [Escherichia albertii TW07627]	343	2E-65

				hypothetical protein VP1802 [Vibrio parahaemolyticus RIMD 2210633]	516	8E-64
				hypothetical protein Maqu_4120 [Marinobacter aquaeolei VT8]	335	4E-49
Xcc_CDS1774	176	3170824 ~ 3171351, (II)	Hypothetical protein	hypothetical protein BPSL0769 [Burkholderia pseudomallei K96243]	167	4E-17
				hypothetical protein amb1133 [Magnetospirillum magneticum AMB-1]	170	3E-16
				hypothetical protein Rsph17025_0806 [Rhodobacter sphaeroides ATCC 17025]	161	1E-06
**Xcc_CDS1783**	970	3201522 ~ 3204431, (I)	Hypothetical protein	hypothetical protein SmalDRAFT_2581 [Stenotrophomonas maltophilia R551-3]	948	0
				hypothetical protein PputW619DRAFT_1952 [Pseudomonas putida W619]	980	1E-133
				val start codon BmulDRAFT_3425 [Burkholderia multivorans ATCC 17616]	990	1E-131
				hypothetical protein BamMC406DRAFT_1773 [Burkholderia ambifaria MC40-6]	937	1E-128
				val start codon CtesDRAFT_0333 [Comamonas testosteroni KF-1]	922	1E-127
				hypothetical protein DaciDRAFT_5892 [Delftia acidovorans SPH-1]	830	1E-115
				hypothetical protein MextDRAFT_0609 [Methylobacterium extorquens PA1]	1243	3E-79
**Xcc_CDS1791**	323	3218354 ~ 3219322, (III)	Hypothetical protein	hypothetical protein Senterenterica_04531 [Salmonella enterica subsp. enterica serovar Agona str. SL483]	127	1E-17
				hypothetical protein CKO_03632 [Citrobacter koseri ATCC BAA-895]	127	1E-17
				hypothetical protein EschericoliO157_07852 [Escherichia coli O157:H7 str. EC4045]	127	3E-17
Xcc_CDS1836	723	3296656 ~ 3294488, (III)	Thermoresistant gluconokinase	putative thermoresistant gluconokinase [Burkholderia thailandensis TXDOH]	687	2E-49
**Xcc_CDS1844**	422	3318234 ~ 3319499, (I)	Conserved hypothetical protein	hypothetical protein SmalDRAFT_1547 [Stenotrophomonas maltophilia R551-3]	767	1E-76
				conserved hypothetical protein PputGB1DRAFT_1223 [Pseudomonas putida GB-1]	400	2E-37
				conserved hypothetical protein BphytDRAFT_3027 [Burkholderia phytofirmans PsJN]	447	1E-27
				conserved hypothetical protein Bcenmc03DRAFT_3615 [Burkholderia cenocepacia MC0-3]	477	2E-26
				conserved hypothetical protein RpicDRAFT_1588 [Ralstonia pickettii 12J]	424	6E-24
				hypothetical protein MpopDRAFT_1789 [Methylobacterium populi BJ001]	452	1E-20
Xcc_CDS1854	62	3339032 ~ 3338847, (III)	Hypothetical protein	hypothetical protein HG1285_16311 [Hydrogenivirga sp. 128-5-R1-1]	61	1E-05
				gp50 [Mycobacterium phage Che9c]	67	2E-05
Xcc_CDS1856	105	3344582 ~ 3344268, (IV)	Hypothetical protein	hypothetical protein XAC1502 [Xanthomonas axonopodis pv. citri str. 306]	306	7E-44
**Xcc_CDS1877**	365	3382105 ~ 3383199, (I)	Conserved hypothetical protein	conserved hypothetical protein [Delftia acidovorans SPH-1]	415	1E-17
				conserved hypothetical protein [Burkholderia ambifaria MC40-6]	431	3E-13
**Xcc_CDS1894**	974	3410909 ~ 3413830, (II)	Conserved hypothetical protein	hypothetical protein SmalDRAFT_1370 [Stenotrophomonas maltophilia R551-3]	553	1E-144
				conserved hypothetical protein SmalDRAFT_1369 [Stenotrophomonas maltophilia R551-3]	470	2E-77
				200 kDa antigen p200, putative [Delftia acidovorans SPH-1]	621	1E-40
				200 kDa antigen p200, putative [Burkholderia ambifaria MC40-6]	535	2E-38
				conserved hypothetical protein BmulDRAFT_2915 [Burkholderia multivorans ATCC 7616]	430	1E-30
				conserved hypothetical protein RpicDRAFT_3259 [Ralstonia pickettii 12J]	503	1E-24
Xcc_CDS1898	103	3419544 ~ 3419236,	Hypothetical protein	hypothetical protein XAC3497 [Xanthomonas axonopodis pv. citri str. 306]	127	7E-13

		(III)		hypothetical protein XOO_2201 [Xanthomonas oryzae pv. oryzae MAFF 311018]	118	8E-05
Xcc_CDS1899	218	3420366 ~3421019, (I)	Thiopurine methyltransferase	thiopurine methyltransferase [Xanthomonas oryzae pv. oryzae KACC10331]	183	6E-55
**Xcc_CDS1916**	363	3465535 ~ 3464447, (III)	Hypothetical protein	hypothetical protein STIAU_6738 [Stigmatella aurantiaca DW4/3-1]	444	4E-26
				hypothetical protein ESA_03155 [Enterobacter sakazakii ATCC BAA-894]	328	1E-24
				hypothetical protein Sententeri_25361 [Salmonella enterica subsp. enterica serovar Dublin str. CT_02021853]	299	6E-23
Xcc_CDS1918	255	3467574 ~ 3466810, (III)	Conserved hypothetical protein	conserved hypothetical protein 701 [Stenotrophomonas maltophilia R551-3]	446	8E-43
Xcc_CDS1947	226	3515430 ~ 3514753, (III)	Hypothetical protein	hypothetical protein Xoryp_15435 [Xanthomonas oryzae pv. oryzicola BLS256]	181	9E-39
				NLP/P60 [Stenotrophomonas maltophilia R551-3]	376	2E-13
**Xcc_CDS2015**	107	3627952 ~ 3627632, (IV)	Putative tryptophan 2,3-dioxygenase oxidoreductase	PUTATIVE TRYPTOPHAN-2,3-DIOXYGENASE OXIDOREDUCTASE PROTEIN [Ralstonia solanacearum GMI1000]	369	1E-28
				Tryptophan 2,3-dioxygenase [Sorangium cellulosum 'So ce 56']	412	1E-26
				putative tryptophan-2,3-dioxygenase oxidoreductase protein [Plesiocystis pacifica SIR-1]	378	2E-19
				predicted protein [Nematostella vectensis]	376	4E-09
**Xcc_CDS2064**	487	3695348 ~ 3696808, (I)	Hypothetical protein	hypothetical protein BmulDRAFT_2069 [Burkholderia multivorans ATCC 17616]	400	4E-35
				hypothetical protein Bcenmc03DRAFT_5820 [Burkholderia cenocepacia MC0-3]	396	1E-33
				conserved hypothetical protein BphytDRAFT_4768 [Burkholderia phytofirmans PsJN]	308	1E-33
Xcc_CDS2133	103	3850565 ~ 3850873, (I)	Hypothetical protein	hypothetical protein Xoryp_19605 [Xanthomonas oryzae pv. oryzicola BLS256]	55	3E-08
				hypothetical protein Xoryp_04800 [Xanthomonas oryzae pv. oryzicola BLS256]	59	6E-08
				hypothetical protein XOO0899 [Xanthomonas oryzae pv. oryzae KACC10331]	60	2E-07
**Xcc_CDS2157**	909	3905752 ~ 3908478, (I)	Conserved hypothetical protein	conserved hypothetical protein [Burkholderia multivorans ATCC 17616]	847	1E-120
				conserved hypothetical protein PputGB1DRAFT_4045 [Pseudomonas putida GB-1]	850	1E-119
				conserved hypothetical protein Bcenmc03DRAFT_4894 [Burkholderia cenocepacia MC0-3]	810	1E-115
				conserved hypothetical protein Rpic12DDRAFT_0659 [Ralstonia pickettii 12D]	784	1E-111
				conserved hypothetical protein MextDRAFT_1857 [Methylobacterium extorquens PA1]	891	1E-111
**Xcc_CDS2161**	291	3918342 ~ 3917470, (III)	Hypothetical protein	hypothetical protein RpicDRAFT_0585 [Ralstonia pickettii 12J]	546	5E-40
				hypothetical protein BamMC406DRAFT_2243 [Burkholderia ambifaria MC40-6]	725	8E-39
				conserved hypothetical protein Bcenmc03DRAFT_4318 [Burkholderia cenocepacia MC0-3]	312	2E-38
Xcc_CDS2168	182	3935066 ~ 3934521, (III)	Hypothetical protein	hypothetical protein XCC0949 [Xanthomonas campestris pv. campestris str. ATCC 33913]	191	1E-29
				hypothetical protein CtesDRAFT_5398 [Comamonas testosteroni KF-1]	196	6E-04
**Xcc_CDS2171**	424	3944066 ~ 3945337, (I)	phenol hydroxylase	phenol hydroxylase XOO3687 [Xanthomonas oryzae pv. oryzae KACC10331]	386	1E-94
				conserved hypothetical protein BphytDRAFT_3233 [Burkholderia phytofirmans PsJN]	396	6E-21
				phenol hydroxylase, putative BmulDRAFT_2806 [Burkholderia multivorans ATCC 17616]	277	1E-17

Xcc_CDS2172	371	3946817 ~ 3947929, (IV)	Conserved hypothetical protein	conserved hypothetical protein DaciDRAFT_5908 [Delftia acidovorans SPH-1]	373	8E-47
				hypothetical protein PputGB1DRAFT_0868 [Pseudomonas putida GB-1]	479	1E-45
				hypothetical protein RpicDRAFT_3186 [Ralstonia pickettii 12J]	390	4E-44
				conserved hypothetical protein CtesDRAFT_1054 [Comamonas testosteroni KF-1]	608	6E-44
				conserved hypothetical protein GbemDRAFT_3026 [Geobacter bemidjiensis Bem]	379	2E-42
**Xcc_CDS2173**	420	3949148 ~ 3947889, (III)	Conserved hypothetical protein	conserved hypothetical protein SmalDRAFT_0763 [Stenotrophomonas maltophilia R551-3]	446	1E-130
				conserved hypothetical protein BphytDRAFT_4764 [Burkholderia phytofirmans PsJN]	422	4E-67
				conserved hypothetical protein BamMC406DRAFT_6083 [Burkholderia ambifaria MC40-6]	460	8E-65
Xcc_CDS2193	359	3972326 ~ 3973402, (I)	Hypothetical protein	hypothetical protein Xoryp_05160 [Xanthomonas oryzae pv. oryzicola BLS256]	125	2E-45
				hypothetical protein EschercoliO157_30609 [Escherichia coli O157:H7 str. EC4042]	126	7E-12
				hypothetical protein SPAB_04257 [Salmonella enterica subsp. enterica serovar Paratyphi B str. SPB7]	126	1E-11
Xcc_CDS2198	189	3976085 ~ 3976651, (I)	Hypothetical protein	hypothetical protein XOO3565 [Xanthomonas oryzae pv. oryzae KACC10331]	191	3E-79
				conserved hypothetical protein EcolBDRAFT_2572 [Escherichia coli B]	183	3E-14
Xcc_CDS2200	294	3977208 ~ 3978089, (I)	Hypothetical protein	hypothetical protein Bpse17_22094 [Burkholderia pseudomallei 1710a]	193	1E-23
				hypothetical protein UTI89_C3752 [Escherichia coli UTI89]	207	8E-11
Xcc_CDS2201	375	3978664 ~ 3979788, (I)	50S ribosomal protein L14	50S ribosomal protein L14 [Stappia aggregata IAM 12614]	147	7E-14
				50S ribosomal protein L14 [Verrucomicrobium spinosum DSM 4136]	130	4E-13
				50S ribosomal protein L14 [Croceibacter atlanticus HTCC2559]	135	7E-13
**Xcc_CDS2202**	133	3979578 ~ 3979976, (I)	Hypothetical protein	hypothetical protein XOO3572 [Xanthomonas oryzae pv. oryzae KACC10331]	300	2E-65
				hypothetical protein NAP1_07385 [Erythrobacter sp. NAP1]	165	6E-28
				hypothetical protein RB2654_11098 [Rhodobacterales bacterium HTCC2654]	173	7E-26
				hypothetical protein RTM1035_02865 [Roseovarius sp. TM1035]	189	5E-25
				hypothetical protein c4074 [Escherichia coli CFT073]	184	7E-23
Xcc_CDS2205	192	3981643 ~ 3982218, (I)	Hypothetical protein	hypothetical protein cgR_5008 [Corynebacterium glutamicum R]	95	4E-07
				hypothetical protein CKO_04728 [Citrobacter koseri ATCC BAA-895]	135	1E-05
				hypothetical protein EcoliO157_00055 [Escherichia coli O157:H7 str. EC4401]	135	5E-05
**Xcc_CDS2206**	309	3982264 ~ 3983190, (I)	Conserved hypothetical protein	conserved hypothetical protein SmalDRAFT_0742 [Stenotrophomonas maltophilia R551-3]	301	7E-75
				conserved hypothetical protein Rpic12DDRAFT_2170 [Ralstonia pickettii 12D]	292	1E-32
				hypothetical protein BamMC406DRAFT_1037 [Burkholderia ambifaria MC40-6]	421	2E-28
				hypothetical protein c4084 [Escherichia coli CFT073]	308	2E-22
**Xcc_CDS2207**	199	3983039 ~ 3983635, (I)	Hypothetical protein	hypothetical protein c4086 [Escherichia coli CFT073]	193	7E-25
				hypothetical protein Sentericaenterica_20570 [Salmonella enterica subsp. enterica serovar Newport str. SL254]	193	1E-24
				hypothetical protein MED92_18513 [Oceanospirillum sp. MED92]	188	2E-18
**Xcc_CDS2208**	309	3983890 ~ 3984816, (I)	Hypothetical protein	hypothetical protein XOO3583 [Xanthomonas oryzae pv. oryzae KACC10331]	309	1E-147
				hypothetical protein MDG893_13819 [Marinobacter algicola DG893]	234	8E-21

				conserved hypothetical protein KT71_01110 [gamma proteobacterium KT 71]	226	4E-18
Xcc_CDS2209	1453	3989969 ~ 3994327, (I)	Hypothetical protein	hypothetical protein GdiaDRAFT_1596 [Gluconacetobacter diazotrophicus PAl 5]	382	2E-71
Xcc_CDS2211	140	3998676 ~ 3999095, (I)	50S ribosomal protein	hypothetical protein CYA_1783 [Synechococcus sp. JA-3-3Ab]	347	8E-08
				hypothetical protein SGO_1190 [Streptococcus gordonii str. Challis substr. CH1]	136	5E-07
				50S ribosomal protein L7/L12 [Streptococcus pneumoniae CGSP14]	133	2E-06
**Xcc_CDS2213**	308	4000399 ~ 4001322, (I)	Hypothetical protein	hypothetical protein XOO3595 [Xanthomonas oryzae pv. oryzae KACC10331]	375	1E-101
				hypothetical protein AvinDRAFT_2599 [Azotobacter vinelandii AvOP]	443	6E-43
				conserved hypothetical protein Bcenmc03DRAFT_3308 [Burkholderia cenocepacia MC0-3]	382	1E-34
Xcc_CDS2236	564	4045360 ~ 4047051, (I)	Hypothetical protein	hypothetical protein STIAU_8083 [Stigmatella aurantiaca DW4/3-1]	579	4E-35
**Xcc_CDS2237**	275	4048579 ~ 4047755, (III)	Hypothetical protein	hypothetical protein PA2G_04052 [Pseudomonas aeruginosa 2192]	243	3E-62
				hypothetical protein EcolBDRAFT_4175 [Escherichia coli B]	217	1E-21
				hypothetical protein ESA_02720 [Enterobacter sakazakii ATCC BAA-894]	171	5E-19
				hypothetical protein MELB17_01515 [Marinobacter sp. ELB17]	177	7E-17
**Xcc_CDS2245**	906	4061565 ~ 4058848, (IV)	Hypothetical protein	hypothetical protein ESA_02657 [Enterobacter sakazakii ATCC BAA-894]	545	5E-73
				hypothetical protein PTH_2866 [Pelotomaculum thermopropionicum SI]	542	6E-63
Xcc_CDS2249	149	4063910 ~ 4064356, (I)	Putative secreted protein	hypothetical protein XOO_3512 [Xanthomonas oryzae pv. oryzae MAFF 311018]	136	2E-39
				putative secreted protein XCV0926 [Xanthomonas campestris pv. vesicatoria str. 85-10]	138	1E-37
				hypothetical protein XAC0891 [Xanthomonas axonopodis pv. citri str. 306]	75	1E-28
Xcc_CDS2263	118	4092224 ~ 4091871, (III)	Hypothetical protein	hypothetical protein GDI0779 [Gluconacetobacter diazotrophicus PAl 5]	185	7E-07
				hypothetical protein c0062 [Escherichia coli CFT073]	117	6E-05
				hypothetical protein EcolO_24846 [Escherichia coli O157:H7 str. EC508]	117	6E-05
**Xcc_CDS2269**	250	4101016 ~ 4101765, (I)	Hypothetical protein	hypothetical protein Bpse14_08382 [Burkholderia pseudomallei 14]	579	3E-05
				hypothetical protein BpseD_08609 [Burkholderia pseudomallei DM98]	579	3E-05
				hypothetical protein BpseN_08019 [Burkholderia pseudomallei NCTC 13177]	579	1E-04
Xcc_CDS2270	348	4106942 ~ 4105899, (III)	Conserved hypothetical protein	conserved hypothetical protein Bcenmc03DRAFT_2858 [Burkholderia cenocepacia MC0-3]	694	2E-63
				hypothetical protein BmulDRAFT_0880 [Burkholderia multivorans ATCC 17616]	342	5E-57
**Xcc_CDS2271**	410	4108226 ~ 4106997, (III)	Conserved hypothetical protein	hypothetical protein BURPS1710b_2031 [Burkholderia pseudomallei 1710b]	1007	1E-55
				conserved hypothetical protein Bcenmc03DRAFT_2858 [Burkholderia cenocepacia MC0-3]	694	4E-47
				conserved hypothetical protein M446DRAFT_0949 [Methylobacterium sp. 4-46]	361	3E-44
**Xcc_CDS2280**	456	4123666 ~ 4122299, (III)	Hypothetical protein	hypothetical protein AvinDRAFT_2299 [Azotobacter vinelandii AvOP]	370	2E-98
				hypothetical protein PY07799 [Plasmodium yoelii yoelii str. 17XNL]	1022	1E-79
				hypothetical protein STIAU_6032 [Stigmatella aurantiaca DW4/3-1]	596	2E-56

Xcc_CDS2291	525	4141965 ~ 4140391, (III)	Hypothetical protein	hypothetical protein PY07799 [Plasmodium yoelii yoelii str. 17XNL]<br>hypothetical protein AvinDRAFT_7247 [Azotobacter vinelandii AvOP]	1022<br>402	1E-35<br>1E-10
Xcc_CDS2303	937	4158099 ~ 4160909, (I)	Hypothetical protein	Hypothetical protein COLAER_01371 [Collinsella aerofaciens ATCC 25986]	955	7E-57
**Xcc_CDS2304**	496	4163645 ~ 4165132, (I)	Hypothetical protein	hypothetical protein Xoryp_04130 [Xanthomonas oryzae pv. oryzicola BLS256]<br>hypothetical protein SBO_0084 [Shigella boydii Sb227]	394<br>432	1E-160<br>1E-46
Xcc_CDS2323	393	4201373 ~ 4202551, (I)	Hypothetical protein	GTP cyclohydrolase/3,4-dihydroxy-2-butanone 4-phosphate synthase bi-functional protein (ribofalvinbiosynthesis) [Rhizobium etli CFN 42]	402	2E-21
**Xcc_CDS2356**	215	4265166 ~ 4265861**,** (II)	Hypothetical protein	hypothetical protein NB231_10593 [Nitrococcus mobilis Nb-231]<br>hypothetical protein N9414_06869 [Nodularia spumigena CCY9414]<br>hypothetical protein Mmc1_0113 [Magnetococcus sp. MC-1]<br>hypothetical protein Mpe_A0008 [Methylibium petroleiphilum PM1]	220<br>220<br>234<br>190	3E-80<br>3E-54<br>4E-53<br>2E-13
Xcc_CDS2363	333	4273417 ~ 4274415, (I)	Hypothetical protein	hypothetical protein STIAU_2095 [Stigmatella aurantiaca DW4/3-1]	647	1E-14
Xcc_CDS2367	172	4282968 ~ 4282453, (III)	Hypothetical protein	hypothetical protein XC_3140 [Xanthomonas campestris pv. campestris str. 8004]<br>hypothetical protein XOO_2943 [Xanthomonas oryzae pv. oryzae MAFF 311018]	98<br>232	1E-17<br>7E-11
**Xcc_CDS2369**	265	4288136 ~ 4288930, (I)	Hypothetical protein	hypothetical protein BthaT_00170 [Burkholderia thailandensis TXDOH]<br>hypothetical protein BoklC_18719 [Burkholderia oklahomensis C6786]<br>hypothetical protein CtesDRAFT_5107 [Comamonas testosteroni KF-1]	642<br>568<br>442	5E-13<br>7E-13<br>9E-11
**Xcc_CDS2371**	293	4296296 ~ 4295418, (III)	Hypothetical protein	hypothetical protein PST_2602 [Pseudomonas stutzeri A1501]<br>conserved hypothetical protein [Rhodopseudomonas palustris TIE-1]<br>hypothetical protein Bpse7_16417 [Burkholderia pseudomallei 7894]<br>hypothetical protein BpseD_16687 [Burkholderia pseudomallei DM98]	599<br>279<br>582<br>582	6E-29<br>1E-28<br>1E-26<br>1E-26
Xcc_CDS2372	349	4297345 ~ 4296299, (III)	Hypothetical protein	hypothetical protein PST_2602 [Pseudomonas stutzeri A1501]<br>hypothetical protein BpseB_15795 [Burkholderia pseudomallei B7210]<br>hypothetical protein Bpse17_31092 [Burkholderia pseudomallei 1710a]	599<br>582<br>582	6E-35<br>3E-17<br>4E-17
**Xcc_CDS2384**	589	4327359 ~ 4325593, (IV)	Hypothetical protein	Hypothetical protein COLAER_01410 [Collinsella aerofaciens ATCC 25986]<br>conserved hypothetical protein [Stigmatella aurantiaca DW4/3-1]<br>hypothetical protein BamMEX5DRAFT_6750 [Burkholderia ambifaria MEX-5]	772<br>611<br>472	2E-31<br>1E-24<br>7E-23
Xcc_CDS2398	116	4359145 ~ 4358798,	Hypothetical protein	unknown protein [Xanthomonas oryzae pv. oryzae KACC10331]	137	4E-27
Xcc_CDS2401	261	4364513 ~ 4365295,	Hypothetical protein	hypothetical protein SARI_03788 [Salmonella enterica subsp. arizonae serovar 62:z4,z23:--]<br>hypothetical protein Sente_26332 [Salmonella enterica subsp. enterica serovar Kentucky str. CVM29188]<br>hypothetical protein Saentericaenterica_11741 [Salmonella enterica subsp. enterica serovar	148<br>148<br>148	1E-08<br>4E-08<br>7E-08

				Newport str. SL317]		
**Xcc_CDS2404**	490	4367860 ~ 4369329, (I)	Conserved hypothetical protein	conserved hypothetical protein SmalDRAFT_3483 [Stenotrophomonas maltophilia R551-3]	489	0
				conserved hypothetical protein AvinDRAFT_8337 [Azotobacter vinelandii AvOP]	586	1E-125
				conserved hypothetical protein RpicDRAFT_4813 [Ralstonia pickettii 12J]	609	1E-121
				conserved hypothetical protein BphytDRAFT_3814 [Burkholderia phytofirmans PsJN]	565	1E-109
				conserved hypothetical protein PputW619DRAFT_3180 [Pseudomonas putida W619]	601	1E-107
				hypothetical protein DaciDRAFT_4309 [Delftia acidovorans SPH-1]	639	5E-96
**Xcc_CDS2412**	646	4377886 ~ 4379299, (I)	Hypothetical protein	hypothetical protein Xoryp_19360 [Xanthomonas oryzae pv. oryzicola BLS256]	724	1E-156
				hypothetical protein ESA_03221 [Enterobacter sakazakii ATCC BAA-894]	702	2E-33
				hypothetical protein SARI_02840 [Salmonella enterica subsp. arizonae serovar 62:z4,z23:--]	644	4E-29
				hypothetical protein CKO_03257 [Citrobacter koseri ATCC BAA-895]	648	3E-26
Xcc_CDS2433	424	4411761~ 4411071, (III)	Hypothetical protein	hypothetical protein XOO0686 [Xanthomonas oryzae pv. oryzae KACC10331]	493	6E-90
**Xcc_CDS2466**	379	4457919 ~ 4457026, (III)	Conserved hypothetical protein	conserved hypothetical protein BphytDRAFT_2313 [Burkholderia phytofirmans PsJN]	378	1E-35
				conserved hypothetical protein BphyDRAFT_5687 [Burkholderia phymatum STM815]	354	9E-33
				conserved hypothetical protein [Delftia acidovorans SPH-1]	339	9E-31
				hypothetical protein DshiDRAFT_0132 [Dinoroseobacter shibae DFL 12]	479	3E-23
**Xcc_CDS2471**	178	4465176 ~ 4464643, (IV)	Hypothetical protein	conserved hypothetical protein [Xanthomonas campestris pv. campestris]	211	1E-31
				hypothetical protein XCV3865 [Xanthomonas campestris pv. vesicatoria str. 85-10]	204	9E-11
				hypothetical protein XAC3746 [Xanthomonas axonopodis pv. citri str. 306]	204	2E-10
**Xcc_CDS2497**	613	4499592 ~ 4501430, (I)	Hypothetical protein	hypothetical protein SmalDRAFT_3538 [Stenotrophomonas maltophilia R551-3]	690	0
				hypothetical protein AvinDRAFT_5400 [Azotobacter vinelandii AvOP]	636	1E-118
				hypothetical protein PputGB1DRAFT_0080 [Pseudomonas putida GB-1]	581	1E-102
				hypothetical protein DaciDRAFT_5863 [Delftia acidovorans SPH-1]	813	1E-101
				hypothetical protein BphyDRAFT_1463 [Burkholderia phymatum STM815]	765	9E-88
**Xcc_CDS2507**	291	4526546 ~ 4527418, (I)	Hypothetical protein	hypothetical protein Rpic12DDRAFT_0493 [Ralstonia pickettii 12D]	393	1E-07
				cell wall surface anchor family protein, putative [Rhodopseudomonas palustris TIE-1]	246	2E-06
Xcc_CDS2516	306	4543746 ~ 4544663, (IV)	Hypothetical protein	hypothetical protein MED222_19443 [Vibrio sp. MED222]	114	1E-11
				hypothetical protein VSWAT3_00095 [Vibrionales bacterium SWAT-3]	116	1E-11
				protein of unknown function DUF1602 [Anaeromyxobacter dehalogenans 2CP-C]	190	1E-10
				hypothetical protein PTD2_18925 [Pseudoalteromonas tunicata D2]	86	2E-09
**Xcc_CDS2518**	630	4549258 ~ 4547945, (III)	Conserved hypothetical protein	hypothetical protein SmalDRAFT_3644 [Stenotrophomonas maltophilia R551-3]	769	1E-137
				conserved hypothetical protein PputGB1DRAFT_0867 [Pseudomonas putida GB-1]	433	6E-91
				conserved hypothetical protein Rpic12DDRAFT_2477 [Ralstonia pickettii 12D]	466	1E-84
				hypothetical protein MchlDRAFT_5004 [Methylobacterium chloromethanicum CM4]	1058	4E-77
				conserved hypothetical protein BphytDRAFT_1848 [Burkholderia phytofirmans PsJN]	398	4E-74
Xcc_CDS2542	229	4592830 ~ 4593516,	Hypothetical protein	hypothetical protein SmalDRAFT_0304 [Stenotrophomonas maltophilia R551-3]	426	2E-41

		(I)		hypothetical protein BthaB_03547 [Burkholderia thailandensis Bt4]	398	3E-18
				hypothetical protein Bpse112_35488 [Burkholderia pseudomallei 112]	436	2E-16
**Xcc_CDS2546**	513	4600388 ~ 4598850, (III)	Putative Atu protein	Atu, putative SmalDRAFT_0303 [Stenotrophomonas maltophilia R551-3]	344	1E-62
				Atu, putative BmulDRAFT_4208 [Burkholderia multivorans ATCC 17616]	485	6E-44
				Atu, putative DaciDRAFT_0923 [Delftia acidovorans SPH-1]	328	1E-33
Xcc_CDS2578	67	4633432 ~ 4633632, (I)	Hypothetical protein	hypothetical protein PROSTU_00109 [Providencia stuartii ATCC 25827]	189	1E-24
				hypothetical protein UTI89_C2919 [Escherichia coli UTI89]	80	2E-24
				conserved hypothetical protein [Escherichia coli APEC O1]	217	2E-24
Xcc_CDS2580	52	4635304 ~ 4635459, (I)	Conserved hypothetical proein	conserved hypothetical protein [Magnetospirillum gryphiswaldense]	259	2E-05
Xcc_CDS2581	79	4635965 ~ 4636201, (I)	Hypothetical protein	hypothetical protein XfasaDRAFT_1247 [Xylella fastidiosa Dixon]	79	5E-37
				hypothetical protein CLOSS21_00494 [Clostridium sp. SS2/1]	63	2E-24
				hypothetical protein BACUNI_00158 [Bacteroides uniformis ATCC 8492]	76	9E-21
				hypothetical protein YintA_01003972 [Yersinia intermedia ATCC 29909]	75	1E-20
Xcc_CDS2582	43	4636553 ~ 4636425, (IV)	Conserved hypothetical protein	Conserved hypothetical protein [Vibrio vulnificus CMCP6]	43	1E-12
				hypothetical protein MCA2723 [Methylococcus capsulatus str. Bath]	43	3E-11
				ORF58e [Pinus koraiensis]	58	3E-08
Xcc_CDS2584	99	4636956 ~ 4637252, (I)	Conserved hypothetical protein	Conserved hypothetical protein [Vibrio vulnificus CMCP6]	76	6E-23
				Conserved hypothetical protein [Vibrio vulnificus CMCP6]	76	2E-22
				hypothetical protein YmolA_01003835 [Yersinia mollaretii ATCC 43969]	65	3E-21
				Orf122 [Chlorobium tepidum]	121	9E-19
				hypothetical protein COXBURSA334_A0040 [Coxiella burnetii RSA 334]	72	1E-17
**Xcc_CDS2589**	428	4638251 ~ 4639534, (I)	Hypothetical protein	hypothetical protein DaciDRAFT_4341 [Delftia acidovorans SPH-1]	505	2E-64
				hypothetical protein BURPS1710b_3413 [Burkholderia pseudomallei 1710b]	823	7E-47
				Hypothetical protein COLAER_01350 [Collinsella aerofaciens ATCC 25986]	425	4E-39
Xcc_CDS2597	1039	4652672 ~ 4655788, (I)	Hypothetical protein	hypothetical protein BthaT_22150 [Burkholderia thailandensis TXDOH]	897	3E-52
				hypothetical protein BpseBC_16497 [Burkholderia pseudomallei BCC215]	897	4E-43
				hypothetical protein BmalP_00725 [Burkholderia mallei PRL-20]	897	1E-42
**Xcc_CDS2599**	421	4661804 ~ 4660542, (III)	Hypothetical protein	protein of unknown function DUF1456 [Stenotrophomonas maltophilia R551-3]	593	3E-59
				hypothetical protein BcenP_01000995 [Burkholderia cenocepacia PC184]	136	5E-07
Xcc_CDS2602	64	4669229 ~ 4669420, (I)	Hypothetical protein	hypothetical protein xccb100_4053 [Xanthomonas campestris pv. campestris]	44	2E-09
Xcc_CDS2612	258	4675197 ~ 4675970, (I)	Hypothetical protein	hypothetical protein BthaT_18800 [Burkholderia thailandensis TXDOH]	132	2E-16
				hypothetical protein Bpse9_06497 [Burkholderia pseudomallei 91]	173	4E-15
				hypothetical protein BpseD_06277 [Burkholderia pseudomallei DM98]	173	5E-15
**Xcc_CDS2653**	664	4726155 ~ 4728146,	Hypothetical protein	hypothetical protein XOO4014 [Xanthomonas oryzae pv. oryzae KACC10331]	662	1E-75

		(I)		hypothetical protein CaulDRAFT_0593 [Caulobacter sp. K31]	532	2E-68
**Xcc_CDS2668**	104	4741139 ~ 4741450, (I)	putative secreted protein	putative secreted protein [Xanthomonas campestris pv. vesicatoria str. 85-10]	104	4E-28
				hypothetical protein SmalDRAFT_0216 [Stenotrophomonas maltophilia R551-3]	147	7E-07
Xcc_CDS2676	250	4749883 ~ 4750595, (I)	Hypothetical protein	hypothetical protein XCV4120 [Xanthomonas campestris pv. vesicatoria str. 85-10]	181	2E-07
				hypothetical protein XOO_0381[Xanthomonas oryzae pv. oryzae MAFF 311018]	226	8E-05
Xcc_CDS2678	404	4755286 ~ 4756427, (I)	WD40-like beta Propeller	WD40-like beta Propeller [Stenotrophomonas maltophilia R551-3]	749	2E-30
**Xcc_CDS2679**	486	4760801~ 4762258, (I)	Hypothetical protein	conserved hypothetical protein 701DaciDRAFT_4304 [Delftia acidovorans SPH-1]	568	1E-71
				hypothetical protein Bcenmc03DRAFT_3311 [Burkholderia cenocepacia MC0-3]	695	6E-63
				hypothetical protein BmulDRAFT_1264 [Burkholderia multivorans ATCC 17616]	650	2E-58
				response regulator receiver domain protein [Stigmatella aurantiaca DW4/3-1]	716	7E-31
**Xcc_CDS2691**	390	4787865 ~ 4786696, (IV)	phenol hydroxylase	phenol hydroxylase [Xanthomonas oryzae pv. oryzae KACC10331]	342	1E-105
				phenol hydroxylase [Xanthomonas axonopodis pv. citri str. 306]	365	9E-16
Xcc_CDS2705	214	4820448 ~ 4819807, (IV)	Hypothetical protein	hypothetical protein xccb100_4194 [Xanthomonas campestris pv. campestris]	214	1E-121
				hypothetical protein SGR_2675 [Streptomyces griseus subsp. griseus NBRC 13350]	210	3E-28
				hypothetical protein STIAU_3940 [Stigmatella aurantiaca DW4/3-1]	219	1E-19
**Xcc_CDS2714**	461	4839502 ~ 4838120, (III)	ferric pseudobactins receptor protein	ferric pseudobactins receptor protein RF5 [Xanthomonas oryzae pv. oryzae KACC10331]	366	6E-79
				hypothetical protein STIAU_8744 [Stigmatella aurantiaca DW4/3-1]	529	2E-17
				ferric pseudobactins receptor protein RF5-like protein [Hahella chejuensis KCTC 2396]	237	4E-15
				ferric pseudobactins receptor protein RF5 Pseudomonas putida (fragment)	173	8E-10
				ferric pseudobactins receptor protein RF5 (ISS) [Ostreococcus tauri]	490	1E-04
**Xcc_CDS2723**	105	4846309 ~ 4846623, (I)	Putative secreted protein	putative secreted protein XCV4247 [Xanthomonas campestris pv. vesicatoria str. 85-10]	120	8E-26
				hypothetical protein XOO_4306 [Xanthomonas oryzae pv. oryzae MAFF 311018]	153	8E-23
Xcc_CDS2750	166	4904900 ~ 4904625, (IV)	Hypothetical protein	hypothetical protein XAC1943 [Xanthomonas axonopodis pv. citri str. 306]	196	2E-16
**Xcc_CDS2766**	142	4943184 ~ 4943609, (II)	Conserved hypothetical protein	hypothetical protein xccb100_4301 [Xanthomonas campestris pv. campestris]	142	1E-76
				hypothetical protein XCV4325 [Xanthomonas campestris pv. vesicatoria str. 85-10]	111	1E-13
				conserved hypothetical protein [Burkholderia graminis C4D1M]	93	2E-06
				hypothetical protein Rmet_6101 [Ralstonia metallidurans CH34]	143	3E-06
Xcc_CDS2803	67	5020819 ~ 5021019, (I)	Conserved hypothetical protein	hypothetical protein PROSTU_00109 [Providencia stuartii ATCC 25827]	189	1E-24
				hypothetical protein UTI89_C2919 [Escherichia coli UTI89]	80	2E-24
				conserved hypothetical protein [Escherichia coli APEC O1]	217	2E-24
Xcc_CDS2805	79	5023352 ~ 5023588, (I)	Hypothetical protein	hypothetical protein XfasaDRAFT_1247 [Xylella fastidiosa Dixon]	79	2E-37
				hypothetical protein YintA_01003972 [Yersinia intermedia ATCC 29909]	75	1E-20
				hypothetical protein BACCAC_03787 [Bacteroides caccae ATCC 43185]	76	2E-18

				hypothetical protein EUBVEN_00028 [Eubacterium ventriosum ATCC 27560]	77	4E-17
				hypothetical protein SpneT_02001730 [Streptococcus pneumoniae TIGR4]	63	4E-16
Xcc_CDS2806	43	5023940 ~ 5023812, (IV)	Hypothetical protein	Conserved hypothetical protein VV2_1439 [Vibrio vulnificus CMCP6]	43	1E-12
				hypothetical protein MCA2723 [Methylococcus capsulatus str. Bath]	43	8E-11
				ORF58e [Pinus koraiensis]	58	3E-08
Xcc_CDS2808	99	5024343 ~ 5024639, (I)	Hypothetical protein	Conserved hypothetical protein VV1_0924 [Vibrio vulnificus CMCP6]	76	7E-24
				hypothetical protein YmolA_01003835 [Yersinia mollaretii ATCC 43969]	65	5E-22
				hypothetical protein Franean1DRAFT_0529 [Frankia sp. EAN1pec]	91	9E-16
Xcc_CDS2820	68	5043917 ~ 5043702, (III)	Hypothetical protein	hypothetical protein XAC1943 [Xanthomonas axonopodis pv. citri str. 306]	196	2E-09
Xcc_CDS2843	121	5065872 ~ 5066234, (I)	Hypothetical protein	unknown protein [Xanthomonas oryzae pv. oryzae KACC10331]	542	3E-15
				unknown protein [Xanthomonas oryzae pv. oryzae KACC10331]	318	4E-15
**Xcc_CDS2849**	307	5071214 ~ 5072134, (I)	Restriction endonuclease	hypothetical protein XCV4427 [Xanthomonas campestris pv. vesicatoria str. 85-10]	171	1E-57
				Restriction endonuclease Noc_A0010 [Nitrosococcus oceani ATCC 19707]	271	4E-34
				putative transmembrane protein MADE_11745 [Alteromonas macleodii 'Deep ecotype']	252	3E-33
				DNA topoisomerase I: Restriction endonuclease [Pseudomonas stutzeri A1501]	298	7E-33
				restriction endonuclease [Delftia acidovorans SPH-1]	428	1E-30
219		(I): 85 (II): 10 (III): 93 (IV): 6				

Additional file 2 - The new CDSs identical to the CDSs annotated in *Xcc* strain B100

New CDSs identified in this study		CDSs annotated in the genome of *Xcc* strain B100		
ID	Size (aa)	ID	Size (aa)	Putative function
**Xcc_CDS002***	285	xccb100_4409	285	Sir2-like protein
Xcc_CDS034	105	xccb100_4461	95	hypothetical protein
Xcc_CDS035	55	xccb100_4462	55	hypothetical protein
**Xcc_CDS073**	123	xccb100_0033	123	peptidase-like protein
Xcc_CDS074	69	xccb100_0034	58	hypothetical protein
Xcc_CDS113	93	xccb100_0076	32	hypothetical protein
**Xcc_CDS141***	186	xccb100_0104	186	hypothetical protein
Xcc_CDS177	77	xccb100_0152	77	IS1480b transposase
**Xcc_CDS356***	142	xccb100_0425	162	putative membrane protein
Xcc_CDS359	79	xccb100_0426	63	hypothetical protein
Xcc_CDS392	90	xccb100_0459	64	hypothetical protein
Xcc_CDS393	87	xccb100_0460	132	hypothetical protein
**Xcc_CDS411***	153	xccb100_0486	228	putative exported protein
Xcc_CDS437	35	xccb100_0519	161	hypothetical protein
Xcc_CDS481	126	xccb100_0574	126	hypothetical protein
Xcc_CDS526	136	xccb100_0662	136	hypothetical protein
Xcc_CDS564	70	xccb100_0714	70	hypothetical protein
**Xcc_CDS577**	140	xccb100_0734	140	hypothetical protein
Xcc_CDS587	299	xccb100_0760	69	hypothetical protein
Xcc_CDS588	38	xccb100_0770	64	hypothetical protein
**Xcc_CDS605**	168	xccb100_0805	168	hypothetical protein
Xcc_CDS606	102	xccb100_0808	80	hypothetical protein
Xcc_CDS688	39	xccb100_4471	39	hypothetical protein
Xcc_CDS746	81	xccb100_1017	81	hypothetical protein
Xcc_CDS806	54	xccb100_1125	54	hypothetical protein
Xcc_CDS896	93	xccb100_1255	88	hypothetical protein
**Xcc_CDS905**	114	xccb100_1266	107	hypothetical protein
Xcc_CDS920	97	xccb100_1278	97	hypothetical protein

Xcc_CDS926	148	xccb100_1279	92	hypothetical protein
Xcc_CDS939	66	xccb100_1301	66	hypothetical protein
**Xcc_CDS961**	139	xccb100_1334	139	hypothetical protein
Xcc_CDS963	117	xccb100_1337	86	hypothetical protein
Xcc_CDS1018	62	xccb100_1433	62	hypothetical protein
**Xcc_CDS1125**	219	xccb100_1603	273	hypothetical protein
**Xcc_CDS1331**	119	xccb100_1997	119	hypothetical protein
**Xcc_CDS1416***	533	xccb100_2400	800	Site-specific DNA-methyltransferase (adenine-specific)
Xcc_CDS1461*	329	xccb100_2347	136	hypothetical protein
Xcc_CDS1480	60	xccb100_2327	96	hypothetical protein
Xcc_CDS1597	82	xccb100_2113	79	hypothetical protein
**Xcc_CDS1698**	129	xccb100_2531	110	hypothetical protein
Xcc_CDS1831	82	xccb100_2772	82	putative exported protein
Xcc_CDS1850	101	xccb100_2813	99	hypothetical protein
**Xcc_CDS1853**	134	xccb100_2816	126	hypothetical protein
Xcc_CDS1854*	62	xccb100_2817	62	hypothetical protein
Xcc_CDS1939	70	xccb100_2979	70	hypothetical protein
**Xcc_CDS1953**	178	xccb100_2996	178	hypothetical protein
Xcc_CDS1989	108	xccb100_3050	108	hypothetical protein
Xcc_CDS2056	66	xccb100_3172	101	hypothetical protein
Xcc_CDS2084	112	xccb100_3232	112	hypothetical protein
Xcc_CDS2085	110	xccb100_3233	110	hypothetical protein
**Xcc_CDS2168***	182	xccb100_3403	182	hypothetical protein
Xcc_CDS2183	74	xccb100_3423	123	hypothetical protein
Xcc_CDS2225	43	xccb100_3499	43	hypothetical protein
**Xcc_CDS2249***	149	xccb100_3535	132	hypothetical protein
**Xcc_CDS2264**	117	xccb100_3568	117	hypothetical protein
Xcc_CDS2293	83	xccb100_3603	83	conserved hypothetical protein
**Xcc_CDS2296**	155	xccb100_3607	179	hypothetical protein
Xcc_CDS2324	94	xccb100_3661	94	putative secreted protein

**Xcc_CDS2356***	215	xccb100_3708	156	hypothetical protein
Xcc_CDS2391	45	xccb100_3770	50	putative secreted protein
Xcc_CDS2545	99	xccb100_4004	109	hypothetical protein
**Xcc_CDS2555**	125	xccb100_0625	127	hypothetical protein
Xcc_CDS2557	46	xccb100_2848	375	hypothetical protein
**Xcc_CDS2668***	104	xccb100_4125	104	Putative secreted protein
Xcc_CDS2688	61	xccb100_4163	48	hypothetical protein
Xcc_CDS2705*	214	xccb100_4194	214	hypothetical protein
**Xcc_CDS2766***	142	xccb100_4301	142	hypothetical protein
Xcc_CDS2769	81	xccb100_4313	74	hypothetical protein
Xcc_CDS2777	77	xccb100_4321	77	small exported protein
Xcc_CDS2799	54	xccb100_4364	54	hypothetical protein
Xcc_CDS2801	39	xccb100_4365	146	hypothetical protein
**Xcc_CDS2849***	307	xccb100_4406	278	hypothetical protein

CDS with detectable transcript by microarray analysis were indicated by bold font. CDS with extrinsic evidence were indicated by *

Additional file 3 - The new CDSs with detectable transcripts by microarray analysis

ID	Size (aa)	Location in the genome of *Xcc* strain 8004	Normalized Signal median[Φ]	Expression pattern[#]
**Xcc_CDS002**	285	5074874 ~ 5075728, (I)	2307 ± 327	b,c
**Xcc_CDS015**	501	5104622 ~ 5106124, (I)	1741 ± 201	a
**Xcc_CDS038**	746	5146091 ~ 5148328, (I)	2572 ± 291	a
Xcc_CDS045	214	11235 ~ 10594, (III)	607 ± 45	b
Xcc_CDS073	123	37084 ~ 36716 (III)	2630 ± 219	a
Xcc_CDS077	245	46007~ 46741, (II)	1690 ± 246	b
**Xcc_CDS103**	323	78431 ~ 79399, (I)	3921 ± 396	a
**Xcc_CDS107**	103	81078 ~ 80770, (III)	5756 ± 1043	a
**Xcc_CDS141**	186	116373 ~ 115816, (III)	957 ± 210	a
**Xcc_CDS150**	383	131241 ~ 132389, (II)	1249 ± 290	b
**Xcc_CDS251**	303	335769 ~ 334861, (III)	3678 ± 321	a
**Xcc_CDS326**	524	431699 ~ 432750, (I)	5621 ± 439	b
**Xcc_CDS333**	515	441103 ~ 439559, (III)	2347 ± 395	a
**Xcc_CDS346**	275	458353 ~ 459177, (I)	175 ± 93	a
**Xcc_CDS356**	142	472327 ~ 471896, (III)	1470 ± 509	b
**Xcc_CDS404**	570	534790 ~ 533033, (III)	903 ± 126	a
**Xcc_CDS411**	153	553624 ~ 553166, (III)	4319 ± 420	b
Xcc_CDS415	381	559732 ~ 558590, (III)	320 ± 490	b
**Xcc_CDS442**	232	601915 ~ 601196, (III)	2928 ± 405	b
Xcc_CDS449	140	616578 ~ 616997, (I)	3007 ± 590	b,c
**Xcc_CDS470**	508	655200 ~ 653677, (III)	2260 ± 293	a
Xcc_CDS488	362	682531 ~ 683622, (I)	2321 ± 510	b
Xcc_CDS489	196	683304 ~ 683891, (I)	3932 ± 467	b,c
**Xcc_CDS538**	801	763903 ~ 766305, (I)	1294 ± 240	a
Xcc_CDS577	140	839802 ~ 840221 (I)	3416 ± 320	b
**Xcc_CDS594**	523	902129 ~ 900465, (III)	2397 ± 321	b
Xcc_CDS605	168	928945 ~ 929448 (I)	4271 ± 395	a
**Xcc_CDS658**	754	1021828 ~1019567, (III)	2239 ± 321	a
Xcc_CDS694	154	1097066 ~ 1097527, (I)	560 ± 364	a
Xcc_CDS700	464	1108378 ~ 1109856, (I)	1214 ± 241	b
Xcc_CDS701	284	1109625 ~ 1110476, (I)	2569 ± 493	b
**Xcc_CDS740**	547	1205358 ~ 1203718, (III)	458 ± 121	a
**Xcc_CDS771**	122	1254035 ~ 1254400, (I)	1732 ± 521	a
Xcc_CDS793	634	1280912 ~ 1279011, (IV)	13 ± 4	a

**Xcc_CDS801**	489	1296479 ~ 1297945, (I)	2382 ± 294	b
Xcc_CDS833	251	1369422 ~ 1370174, (I)	4791 ± 407	b,c
**Xcc_CDS834**	600	1372065 ~ 1370266, (III)	3934 ± 320	a
**Xcc_CDS839**	884	1378682 ~ 1376031, (III)	7321 ± 532	a
**Xcc_CDS842**	254	1385415 ~ 1384654, III)	4209 ± 292	b
**Xcc_CDS866**	229	1403462 ~ 1404148, (I)	8900 ± 432	b
Xcc_CDS905	114	1496595 ~ 1496936 (III)	3207 ± 190	a
**Xcc_CDS936**	605	1528313 ~ 1530127, (I)	3894 ± 290	a
**Xcc_CDS947**	436	1554197~ 1555504, (I)	4761 ± 310	b
Xcc_CDS961	139	1577011 ~ 1576595 (III)	4390 ± 291	a
Xcc_CDS976	420	1593446 ~ 1594705, (I)	1932 ± 200	b,c
Xcc_CDS1011	229	1665418 ~ 1664732, (III)	6231 ± 432	a
Xcc_CDS1025	276	1679672 ~ 1678845, (III)	3789 ± 421	a
**Xcc_CDS1047**	278	1715471 ~ 1714638, (III)	2893 ± 320	a
**Xcc_CDS1079**	638	1775688 ~ 1773775, (III)	5417 ± 323	a
**Xcc_CDS1095**	1283	1804264 ~ 1800416, (III)	2475 ± 389	b
**Xcc_CDS1100**	537	1823106 ~ 1821496, (III)	4921 ± 327	a
Xcc_CDS1125	219	1877142 ~ 1876486 (IV)	2948 ± 219	b
**Xcc_CDS1129**	415	1885910 ~ 1884642 , (III)	9354 ± 619	b
**Xcc_CDS1147**	784	1922714 ~ 1920363, (III)	3964 ± 392	b
**Xcc_CDS1152**	767	1932962 ~ 1930662, (III)	3789 ± 173	b
**Xcc_CDS1183**	797	1992481 ~ 1990091, (III)	2895 ± 213	a
Xcc_CDS1210	140	2056355 ~ 2056807, (III)	2070 ± 419	a
**Xcc_CDS1224**	384	2098936 ~ 2097785, (III)	4167 ± 290	b
**Xcc_CDS1274**	495	2219040 ~ 2220524, (IV)	6783 ± 395	a
**Xcc_CDS1309**	424	2294965 ~ 2294112, (III)	4534 ± 278	a
**Xcc_CDS1324**	601	2324852 ~ 2323050, (III)	7413 ± 923	b,c
Xcc_CDS1331	119	2335517 ~ 2335873, (II)	3210 ± 301	b
**Xcc_CDS1340**	502	2351256 ~ 2352761, (I)	7023 ± 194	a
**Xcc_CDS1381**	181	2428687 ~ 2428145, (III)	3962 ± 891	a
**Xcc_CDS1402**	283	2470019 ~ 2469171, (IV)	4321 ± 380	b
**Xcc_CDS1416**	533	2489339 ~ 2490937, (II)	6203 ± 249	a
**Xcc_CDS1436**	1272	2521043 ~ 2524858, (I)	3207 ± 213	a
**Xcc_CDS1499**	986	2622928 ~ 2625885, (I)	4291 ± 460	b
Xcc_CDS1524	465	2695669 ~ 2694275, (III)	3637 ± 541	b
Xcc_CDS1545	140	2731355 ~ 2730908, (III)	8572 ± 732	a
**Xcc_CDS1553**	122	2764864 ~ 2764517, (IV)	4209 ± 218	b
**Xcc_CDS1561**	689	2775992 ~ 2774779, (III)	3789 ± 401	a

**Xcc_CDS1569**	537	2787422 ~ 2785811, (III)	3219 ± 219	a
**Xcc_CDS1585**	531	2824681~ 2826273, (I)	5631 ± 297	b
**Xcc_CDS1588**	427	2842397 ~ 2843677, (I)	2914 ± 930	a
**Xcc_CDS1604**	282	2867174 ~ 2868019, (I)	4204 ± 263	b
Xcc_CDS1608	394	2872910 ~ 2874091, (I)	4352 ± 521	b, c
**Xcc_CDS1619**	245	2886681 ~ 2885857, (III)	2974 ± 213	b
**Xcc_CDS1633**	117	2926523 ~ 2926173, (III)	5690 ± 423	b
Xcc_CDS1698	129	3031983 ~ 3031597 (III)	6495 ± 395	a
**Xcc_CDS1718**	409	3061241 ~ 3062467, (I)	6799 ± 398	a
**Xcc_CDS1719**	298	3062814 ~ 3063707, (I)	8532 ± 539	b
**Xcc_CDS1746**	134	3122564 ~ 3122037, (III)	2994 ± 212	a
**Xcc_CDS1757**	141	3140508 ~ 3140952, (I)	2348 ± 204	b,c
**Xcc_CDS1770**	285	3164786 ~ 3163932, (III)	3974 ± 219	a
**Xcc_CDS1783**	970	3201522 ~ 3204431, (I)	2325 ± 401	b
Xcc_CDS1784	655	3204431 ~ 3206395, (I)	5218 ± 620	b,c
**Xcc_CDS1791**	323	3218354 ~ 3219322, (III)	4967 ± 329	a
Xcc_CDS1811	457	3252432 ~ 3251062, (III)	2432 ± 379	a
**Xcc_CDS1844**	422	3318234 ~ 3319499 , (I)	3957 ± 230	b
Xcc_CDS1853	134	3338871 ~ 3338470, (III)	9472 ± 621	b
**Xcc_CDS1877**	365	3382105 ~ 3383199, (I)	3193 ± 194	a
**Xcc_CDS1894**	974	3410909 ~ 3413830, (II)	7490 ± 239	a
**Xcc_CDS1916**	363	3465535 ~ 3464447, (III)	4321 ± 294	a
Xcc_CDS1953	178	3519616 ~ 3520149, (I)	8476 ± 567	b
Xcc_CDS1991	258	3587959 ~ 3586973, (III)	6273 ± 487	b,c
**Xcc_CDS2015**	107	3627952 ~ 3627632, (IV)	2479 ± 507	a
**Xcc_CDS2064**	487	3695348 ~ 3696808, (I)	3294 ± 295	a
**Xcc_CDS2157**	909	3905752 ~ 3908478, (I)	8390 ± 431	a
**Xcc_CDS2161**	291	3918342 ~ 3917470, (III)	4319 ± 307	a
**Xcc_CDS2168**	182	3935066 ~ 3934521, (III)	6890 ± 346	b
**Xcc_CDS2171**	424	3944066 ~ 3945337, (I)	9210 ± 379	a
**Xcc_CDS2173**	420	3949148 ~ 3947889, (III)	1898 ± 1210	b
**Xcc_CDS2202**	133	3979578 ~ 3979976, (I)	7217 ± 596	a
**Xcc_CDS2206**	309	3982264 ~ 3983190 , (I)	5832 ± 317	b
**Xcc_CDS2207**	199	3983039 ~ 3983635, (I)	6541 ± 295	a
**Xcc_CDS2208**	309	3983890 ~ 3984816, (I)	2432 ± 293	b
**Xcc_CDS2213**	308	4000399 ~ 4001322, (I)	7463 ± 396	b
**Xcc_CDS2237**	275	4048579 ~ 4047755, (III)	2907 ± 231	b
**Xcc_CDS2245**	906	4061565 ~ 4058848, (IV)	3949 ± 215	a

**Xcc_CDS2249**	149	4063910 ~ 4064356, (I)	8734 ± 263	b
Xcc_CDS2264	117	4097319 ~ 4096969, (IV)	2340 ± 947	b,c
**Xcc_CDS2269**	250	4101016 ~ 4101765, (I)	4321 ± 294	a
**Xcc_CDS2271**	410	4108226 ~ 4106997, (III)	6043 ± 387	a
**Xcc_CDS2280**	456	4123666 ~ 4122299, (III)	5321 ± 423	b
Xcc_CDS2292	109	4141997 ~ 4142323, (I)	7732 ± 431	a
Xcc_CDS2296	155	4144739 ~ 4144275, (IV)	2965 ± 201	a
Xcc_CDS2299	153	4151875 ~ 4151414, (III)	1873 ± 159	a
**Xcc_CDS2304**	496	4163645 ~ 4165132, (I)	9481 ± 529	a
Xcc_CDS2308	506	4171094 ~ 4172611, (I)	4961 ± 310	b,c
Xcc_CDS2336	71	4230234 ~ 4230022, (III)	5793 ± 321	b,c
**Xcc_CDS2356**	215	4265166 ~ 4265861, (II)	1974 ± 590	b,c
**Xcc_CDS2369**	265	4288136 ~ 4288930, (I)	7541 ± 510	a
**Xcc_CDS2371**	293	4296296 ~ 4295418, (III)	3986 ± 201	b
Xcc_CDS2379	263	4305818 ~ 4306774, (I)	7219 ± 493	b, c
**Xcc_CDS2384**	589	4327359 ~ 4325593, (IV)	4946 ± 320	a
**Xcc_CDS2404**	490	4367860 ~ 4369329, (I)	6271 ± 320	b
**Xcc_CDS2412**	646	4377886 ~ 4379299, (I)	8903 ± 531	a
Xcc_CDS2445	492	4426252 ~ 4424777, (IV)	1921 ± 213	a
**Xcc_CDS2466**	379	4457919 ~ 4457026, (III)	5703 ± 389	b
**Xcc_CDS2471**	178	4465176 ~ 4464643, (IV)	7431 ± 307	b
**Xcc_CDS2497**	613	4499592 ~ 4501430, (I)	4987 ± 193	d
**Xcc_CDS2507**	291	4526546 ~ 4527418, (I)	4936 ± 320	a
**Xcc_CDS2518**	630	4549258 ~ 4547945, (III)	9784 ± 421	a
**Xcc_CDS2546**	513	4600388 ~ 4598850, (III)	8340 ± 467	a
Xcc_CDS2555	125	4616124 ~ 4615750, (III)	6531 ± 421	b
**Xcc_CDS2589**	428	4638251 ~ 4639534, (I)	8467 ± 430	a
**Xcc_CDS2599**	421	4661804 ~ 4660542, (III)	6407 ± 453	b
**Xcc_CDS2653**	664	4726155 ~ 4728146, (I)	4967 ± 286	a
**Xcc_CDS2668**	104	4741139 ~ 4741450, (I)	4067 ± 401	b
**Xcc_CDS2679**	486	4760801~ 4762258, (I)	8320 ± 395	b
**Xcc_CDS2691**	390	4787865 ~ 4786696, (IV)	5021 ± 307	a
**Xcc_CDS2714**	461	4839502 ~ 4838120, (III)	4201± 307	b
**Xcc_CDS2723**	105	4846309 ~ 4846623, (I)	3095 ± 105	b
**Xcc_CDS2766**	142	4943184 ~ 4943609, (II)	6596 ± 395	a
Xcc_CDS2824	370	5048911 ~ 5047802, (III)	1994 ± 347	a
**Xcc_CDS2849**	307	5071214 ~ 5072134, (I)	4992 ± 294	b

The CDS with extrinsic evidence were indicated by bold fonts.

Φ The normalized signal medians presented in this table are the highest ones among all the results from different experiments.# (a) constitutive expression; (b) high cell density-dependent; (c) DSF signal-regulated; (d) NYG medium-induced.

Additional file 4- Oligos used in this study

Application	Oligos and sequences (5' to 3')	
RT-PCR analysis	Xcc_CDS002	2-RT-FOR: CCC TTG GAA CCG ATA ATA AAA CT
		2-RT-REV: ATA GAC GGC GCT GGA CTG AGA
	Xcc_CDS449	449-FOR: CAG CTG GAC CGC GTG TAT TGC
		449-REV: AGC ATT CCT CCC GAT ACCTA
	Xcc_CDS489	489-FOR: CGG TGC GCA GGC TCA GGT GT
		489-REV: GCC GAC GGT TGG GTG CTC A
	Xcc_CDS1324	1324-FOR: ATG CGG CGA CGC TCT TCA TC
		1324-REV: CGG CGC ATT CGT GGA CCT G
	Xcc_CDS1608	1608-FOR: GAT GCC TGC TCG TCC AAC TCG T
		1608-REV: GGC CTT CCT GCG TGA CCT GTG
	Xcc_CDS1757	1757-FOR: GGG CGT GTA TCG ATG AAG GAG AAG
		1757-REV: GGG GCA GGT CAC CGG GAA TCA GC
	Xcc_CDS1784	1784-FOR: CAG CAG CGG ACG GAT GTG GT
		1784-REV: GGT GTG GCC GTG CAA GGA AGG
	Xcc_CDS2264	2264-FOR: CAG TGG TGG GCA GCG AGT AGA TGG
		2264-REV: CCC TGC GTG GCG GCT GAC CTG
	Xcc_CDS2336	2336FOR: ATA GTG GCA TAC CGC TTT CA
		2336REV: AGG ATC CTG CCA CCA AGT AT
	Xcc_CDS2356	2356-FOR: TGA ACC AGC TTC CCA GAC TCC
		2356-REV: TGC AGA TCC GCC AGG GTG AAC
	Xcc_CDS2379	2379FOR: TCG ATG TCG TGG CCG GTG ATT TTC
		2379REV: ACC GAC GAG TTC TGG CCG ATG GAT
	16S rDNA	16S-F: GTTCCCGAAGGCACCAATCCATCT
		16S-R: GGGCGTAAAGCGTGCGTAGGTG
CDS deletion	*sir2x*	FOR1: AAA AAC TGG ACC AAG GGG ATA GAA AG
		REV1: TCATT CAA CGC AAC ACC GAA CCA AGT AGG AGG GCG AGT TTT
		FOR2: CCT TGG CCG CGA CTA TGA CGA CA
		REV2: CCT TGG CCG CGA CTA TGA CGA CA
	arsR	FOR1: AA AGG CGC CTT TTT GCT GAC TGG
		REV1: TGG CTC CGG CTG ACA TTC CTT CC
		FOR2: AGG AAT GTC AGC CGG AGC CA GCT CCT GCC TTT CGC CCT CAAT
		REV2: GCA AAC CTG CGC GTA AAT CTC CTT
Complementation	*sir2x*	FOR: CGGGATCC GTG AAA GTG ATA GAT TGG CCA
		REV: CCC AAG CTT TCA ATT TCT TGC CAG ATT CAC CTT
	arsR	FOR: CG GGATCC ATG GAA GAG CAT GCA GCG TTG
		REV: CCC AAG CTT TCA CCG AGG CAA CTC GTC CGT AG
Operon analysis	*Sir2x* operon	F1: TTCCCGGCGACCCTAAGTTTTCTC
		R1: ATTCCGGAT ATTACGCTGTCAAAC
		R2: TGGGGTAGTTATGCTTCCGTGTAG
	arsR operon	F1: CGC TTT CTT GAT GCA TGA CTG TTG
		R1: GGG GAT TGG ATA GGC ACG GAT TAG
		R2: AAG CGA GCG ATT GGC ACC TGA AGA
